# Cwh8 moonlights as a farnesyl pyrophosphate phosphatase and is essential for farnesol biosynthesis in *Candida albicans*

**DOI:** 10.1128/mbio.02290-25

**Published:** 2025-09-08

**Authors:** Daniel J. Gutzmann, Cory H. T. Boone, Brigid M. Toomey, Shyanne Urbin, Wayne R. Riekhof, Deborah A. Hogan, Audrey L. Atkin, Kenneth W. Nickerson

**Affiliations:** 1 School of Biological Sciences, University of Nebraska-Lincoln 14719, Lincoln, Nebraska, USA; 2 Department of Microbiology and Immunology, Geisel School of Medicine at Dartmouth 12285, Hanover, New Hampshire, USA; Instituto Carlos Chagas, Curitiba, Brazil

**Keywords:** *Candida albicans*, farnesol biosynthesis

## Abstract

In the opportunistic pathogen *Candida albicans*, hyphal growth and virulence factor expression are regulated by environmental and chemical cues. Farnesol is a secreted autoregulatory molecule that represses filamentation. It is derived from farnesyl pyrophosphate (FPP), an ergosterol biosynthesis pathway intermediate. Although Dpp1, Dpp2, and Dpp3 were proposed to synthesize farnesol, a mutant lacking all three had only a modest reduction in farnesol. To identify other farnesol biosynthesis genes, we employed transcription factor mutants that underproduced or overproduced farnesol in a screen and analyzed their transcriptomes. *CWH8* was the only transcript correlated with farnesol production. Cwh8 is a lipid phosphatase known to recycle dolichyl pyrophosphate during glycosylation of cell wall proteins. The *cwh8ΔΔ* mutant had a > 99% reduction in farnesol compared to the parent strain and was hyperfilamentous in embedded conditions. It had ~900 fold lower levels of farnesyl phosphate, the product of FPP dephosphorylation, suggesting Cwh8 acts directly on FPP. Complementation of the *C. albicans cwh8ΔΔ* mutant with *CWH8* from *C. albicans* restored cell wall integrity and farnesol production. However, complementation with *CWH8* from *Clavispora lusitaniae*, a species lacking farnesol signaling, restored cell wall integrity but failed to rescue farnesol production. This indicates that while Cwh8’s role in maintaining cell wall integrity is conserved, differences in substrate specificity or interactions with species-specific cofactors underlie its involvement in farnesol biosynthesis in *C. albicans*. Finally, fluconazole was fungicidal for *cwh8ΔΔ* rather than fungistatic. These studies demonstrate how an extracellular signaling system can arise and be co-opted to promote fungal fitness in complex environments.

**IMPORTANCE:**

Farnesol secretion distinguishes the human opportunistic pathogen *Candida albicans* from non-secreting, non-pathogenic yeasts. Despite 20 years of research, surprisingly little is known about how farnesol is synthesized and regulated. Using transcriptomic profiles from mutants with altered farnesol production, we identified *CWH8* as a critical enzyme in this process. *CWH8* null mutants produced no farnesol. Our novel assay measuring cellular farnesyl pyrophosphate (FPP) and farnesyl phosphate (FP) showed that Cwh8 converts FPP to FP in the first step of farnesol biosynthesis, in addition to its established role in recycling dolichyl pyrophosphate. Farnesol-secreting fungi had huge metabolic pools of FP, while nonsecretors had small pools. Furthermore, expressing *CWH8* from *Clavispora lusitaniae*, a non-farnesol-secreting species, failed to restore farnesol production in *C. albicans*. This suggests that changes in Cwh8 enzyme specificity drove the evolution of farnesol as a signaling molecule and virulence factor in *C. albicans*.

## INTRODUCTION

**Fig 1:**
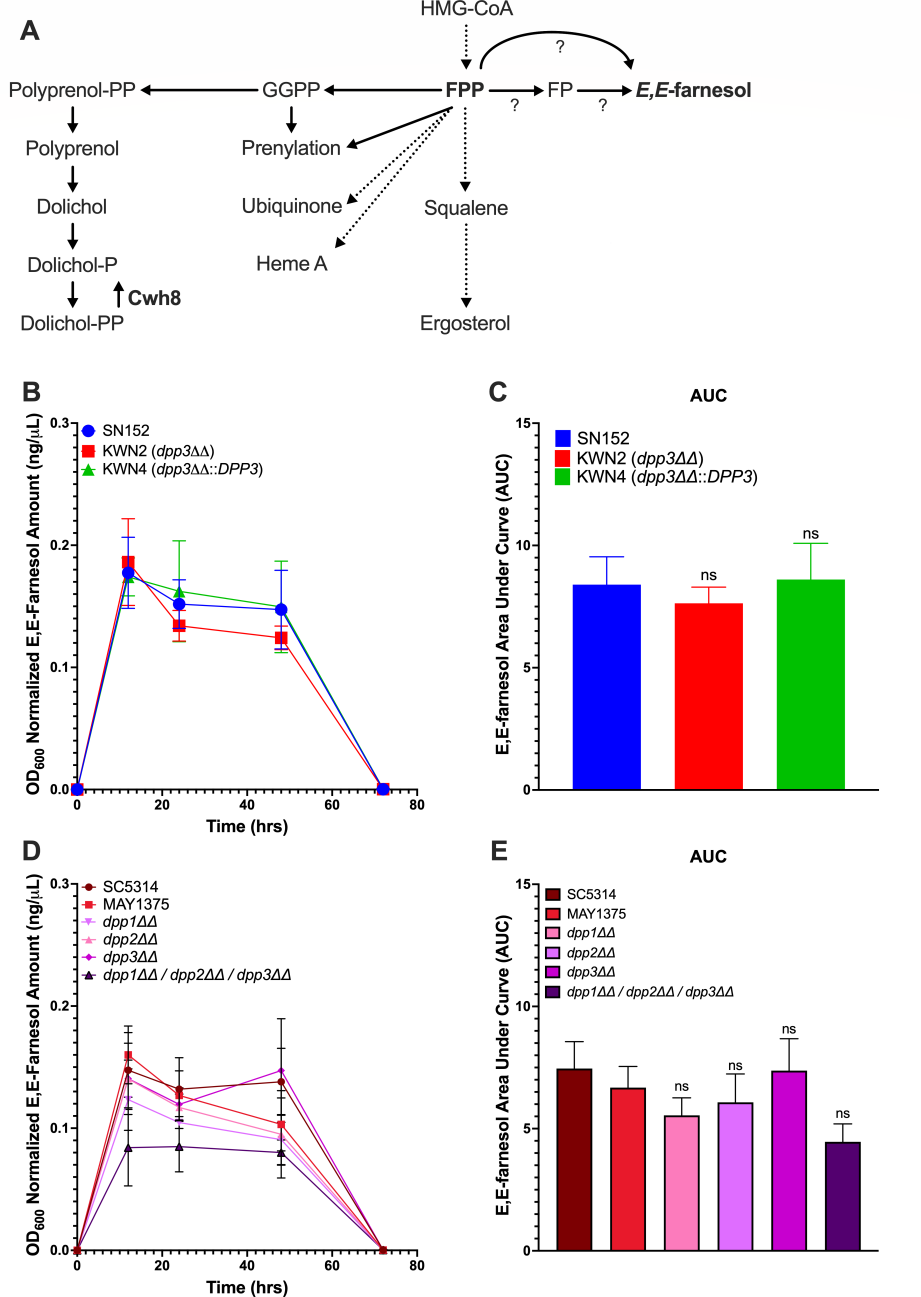
Dpp1, Dpp2, and Dpp3 do not account for all the farnesyl pyrophosphatase activity in *C. albicans*. (**A**) Proposed model for farnesol biosynthesis. (**B**) Farnesol accumulation assessed at 12, 24, 48, and 72 h post-inoculation at 30°C in YPD for SN152, KWN2 (*dpp3ΔΔ*), and KWN4 (*dpp3ΔΔ::DPP3*) or (**D**) SC5314, MAY1375*, dpp1ΔΔ, dpp2ΔΔ, dpp3ΔΔ, and dpp1ΔΔ/dpp2ΔΔ/dpp3ΔΔ*. Data are the mean OD_600_ normalized whole farnesol accumulation ±SEM of three independent growth curves. Area under the curve (AUC) analyses (**C**) and (**E**) were performed on the curves presented in (**B**) and (**D**), and data are the mean AUC ±SEM of three independent growth curves. Differences between groups were determined by one-way ANOVA with Dunnett’s multiple comparisons test. Differences were considered significant at *P* < 0.05.

** **The opportunistic commensal, *Candida albicans,* is a polymorphic fungus that resides primarily in the gastrointestinal and genitourinary tracts of 40%–60% of healthy individuals (1–3). In patients with compromised immune systems, *C. albicans* can disseminate through the intestinal mucosa or via implanted devices, leading to life-threatening invasive infections with mortality rates of up to 64% (4–6). A key trait that governs invasion and dissemination is morphogenic plasticity (7) in which *C. albicans* switches between yeast and filamentous morphologies in response to environmental factors (8, 9). Growth in the hyphal morphology is strongly associated with *C. albicans* virulence (5, 10, 11), and farnesol (12), a *C. albicans* secreted autoregulatory molecule (13), is an important repressor of hyphal growth. Farnesol is a secondary metabolite derived from the ergosterol biosynthesis intermediate farnesyl pyrophosphate (FPP); see Fig. 1A. In addition to inhibiting the yeast-hyphae transition, hyphal extension, and biofilm formation (12, 14–16), farnesol can cause damage during systemic infection (17–19), modulate interactions with host immune cells (17, 18, 20, 21), and act as an antimicrobial (22–25). These bioactivities have been extensively reviewed (26, 27). Despite these advances, the precise mechanisms for the synthesis, secretion, regulation, and turnover of farnesol are poorly understood.

Dpp1, Dpp2, and Dpp3 were published as FPP phosphatases in *C. albicans* (28). *C. albicans* Dpp2 and Dpp3 are orthologs of the *Saccharomyces cerevisiae* Lpp1 and Dpp1 lipid phosphate phosphatases. In addition to catalyzing the dephosphorylation of phosphatidic acid (PA) to form diacylglycerol (DAG) (29), biochemical studies showed that Lpp1 and/or Dpp1 could act on isoprenoid phosphates including dolichyl phosphate (Dol-P), dolichyl pyrophosphate (Dol-PP), farnesyl pyrophosphate (FPP), and geranylgeranyl pyrophosphate (GG-PP) (29, 30). Navarathna et al. (19) examined a *dpp3* null mutant in *C. albicans* strain SN152 background and found that it accumulated ~17% of the farnesol of its parent. However, Polke et al. (31) later generated a *dpp3* null mutant in *C. albicans* strain SC5314 and found no decrease in farnesol accumulation, suggesting that the contribution of Dpp3 may be strain-specific and/or dispensable for farnesol synthesis in *C. albicans* (26, 31).

In this study, we sought to identify factors that are involved in the synthesis of farnesol in *C. albicans*. We first showed that the activities of Dpp1, Dpp2, and Dpp3 do not account for much of the farnesol synthesis. Using a genetic screen for farnesol overproducers and underproducers combined with transcriptomics, we identified one gene with an expression pattern that correlated with farnesol production: *CWH8*. Using genetics and metabolite analyses, we demonstrate Cwh8 is a novel FPP phosphatase necessary for most of the farnesol production in *C. albicans*. Cwh8 has been previously characterized as a dolichyl-pyrophosphate phosphatase (32) that regenerates Dol-P from Dol-PP for use as a glycosyl carrier lipid during N-glycosylation of proteins (32) as well as the synthesis of glycoproteins, GPI-anchored proteins, and cell wall mannans (32–34), and our data suggest that *C. albicans* Cwh8 may have evolved another function in farnesol production.

## RESULTS

### Dpp1, Dpp2, and Dpp3 make only a minor contribution to farnesol synthesis in *C. albicans*

**Fig 2:**
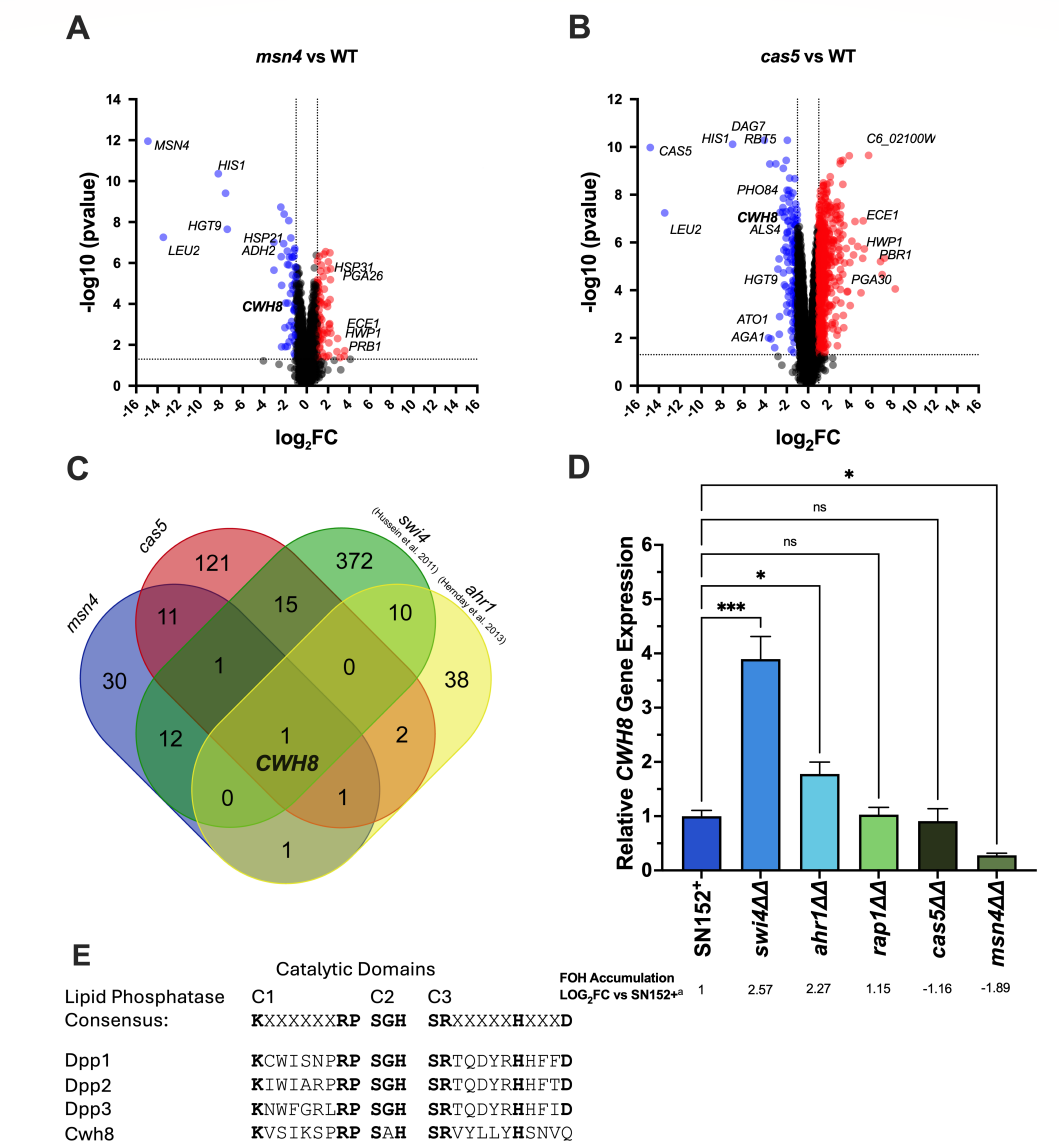
RNA-seq analysis on farnesol underproducing mutants (*msn4ΔΔ* and *cas5ΔΔ*) identifies *CWH8* as a candidate gene involved in farnesol production. Volcano plots of differentially expressed genes in the *msn4ΔΔ* (**A**) and *cas5ΔΔ* (**B**) mutants. (**C**) Gene overlap analysis of the differentially downregulated genes in the farnesol under accumulating mutants *cas5ΔΔ* and *msn4ΔΔ* with those differentially upregulated in the farnesol-overproducing mutants *swi4ΔΔ* and *ahr1ΔΔ*. (**D**) RT-qPCR analysis of *CWH8* gene expression in five transcription regulator deletion mutants with farnesol accumulation differences. Data are mean *CWH8* expression ±SD relative to the paired parent strain (SN152^+^) of three independent experiments. Differences between groups were determined by one-way ANOVA with Dunnett’s multiple comparisons test. Differences were considered significant at *P* < 0.05 (*P < 0.05, **P < 0.01, and ***P < 0.001). (**E**) Protein sequence alignment of the lipid phosphatase catalytic domains of the putative farnesyl pyrophosphatases in *C. albicans*.

Identifying the genes involved in the conversion of FPP to farnesol has been difficult (Fig. 1A). For a long time, Dpp1, Dpp2, and Dpp3 were the presumed FPP phosphatases (35). These results seemed reasonable. Dpp2 and Dpp3 were chosen because they have the consensus lipid phosphatase catalytic domains (see Fig. 2E) as well as having homologs in *S. cerevisiae* known to encode broad-specificity pyrophosphatases able to use FPP and GGPP as substrates in *in vitro* assays (29). Dpp1 does not have a precise ortholog in *S. cerevisiae* but was identified as a third possible FPP pyrophosphatase because it too had the consensus lipid phosphatase catalytic domain. To clarify these conflicting reports on the role of Dpp3 in farnesol biosynthesis (19, 31), we generated a set of *dpp1ΔΔ*, *dpp2ΔΔ*, and *dpp3ΔΔ* null mutants alone and in combination (36). Farnesol production by four mutants and their MAY1375 parent, as well as the *dpp3ΔΔ* null mutant generated previously by our group (19), was examined at intervals over 72 hours looking for changes in farnesol synthesis, turnover, or decay (Fig. 1). Throughout, we used the improved gas chromatography assay described by Boone et al. (13). The triplicate values we report now are the whole culture accumulation values because at no point did we discover significant differences in the cell pellet/supernatant ratios for these strains. Measurements at 12, 24, 48, and 72 hours (Fig. 1B and D) were integrated and reported as areas under the curve (AUC) (Fig. 1C and E). The *dpp3ΔΔ* mutant (KWN2) developed previously had only a 9.2% reduction in total farnesol synthesis (Fig. 1C). There were no differences at 12 hours while the greatest differences were seen at 48 hours (Fig. 1B), suggesting an effect on farnesol turnover or decay rather than farnesol synthesis.

The newly generated *dpp1ΔΔ, dpp2ΔΔ,* and *dpp3ΔΔ* single mutants displayed modest reductions in farnesol accumulation when compared with their MAY1375 parent (Fig. 1D and E). When grown in YPD, their total farnesol production values (AUC) were reduced by ca. 17, 9, and 0%, respectively (Fig. 1E). The greatest loss of farnesol accumulation occurred when *DPP1* was absent (Fig. 1E). For the *dpp1ΔΔ/dpp2ΔΔ/dpp3ΔΔ* triple mutant, the total AUC production was reduced by 33% (Fig. 1E). These results suggest that Dpp1, Dpp2, and Dpp3 account for only a small portion of the FPP phosphatase activity in *C. albicans*.

### Analysis of transcriptomic profiles identifies *CWH8* as a candidate gene for farnesol synthesis

To identify other genes involved in the synthesis of farnesol, we leveraged a recent screen of 165 mutants of the Homann transcription regulator knockout collection (37) for differences in their farnesol accumulation and supernatant/pellet localization (38). Nineteen of the 165 mutants showed significant differences, with ten mutants producing more farnesol than their SN152^+^ control strain, while nine produced less (38). The transcriptomic profiles of the two highest producing deletion mutants, *swi4ΔΔ* and *ahr1ΔΔ* (38), have previously been characterized (39, 40). We identified 11 genes whose transcripts were significantly higher in both mutants (39, 40): they involved membrane transport (*SFC1*), metabolism (*BIO2*, *FDH1*, *SOD3*, and *ALD6*), DNA damage (*RFX2*), morphogenesis (*SFL2*), and cell wall structure and function (*RBT1, CRH11, PGA13, and CWH8*). For the farnesol underproducing transcription factor mutants, we performed RNA-seq analysis of *msn4ΔΔ* and *cas5ΔΔ* in comparison to their parental strain (Fig. 2A and B). Using conventional thresholds of an adjusted *P*-value < 0.05 and a log_2_ fold change >1 (corresponding to a twofold increase or decrease), we identified 124 and 648 differentially expressed genes in the *msn4ΔΔ* and *cas5ΔΔ* mutants, respectively (Fig. 2A and B; Table S2), including 57 and 152 downregulated genes in *msn4ΔΔ* and *cas5ΔΔ,* respectively, with 14 of those genes being downregulated in both (Fig. 2C). We next compared the lists of differentially downregulated genes in the *msn4ΔΔ* and *cas5ΔΔ* transcriptomes with the list of differentially upregulated genes in the transcriptomes of *swi4ΔΔ* and *ahr1ΔΔ* and found that *CWH8,* which encodes a dolichyl-PP phosphatase, was the only one present on all four lists (Fig. 2C). To validate the RNA-seq and microarray data sets, we performed RT-qPCR for *CWH8* gene expression in these four mutants along with *rap1ΔΔ,* another farnesol overproducer (38). As expected, *CWH8* was upregulated in *swi4ΔΔ* and *ahr1Δ* and downregulated in *msn4ΔΔ* (Fig. 2D). However, we did not detect a statistically significant change in *CWH8* gene expression in either *cas5ΔΔ* or *rap1ΔΔ* (Fig. 2D), suggesting that other mechanisms for the regulation of farnesol production exist.

### Cwh8 is a farnesyl pyrophosphate phosphatase necessary for farnesol synthesis in *C. albicans*

**Fig 3:**
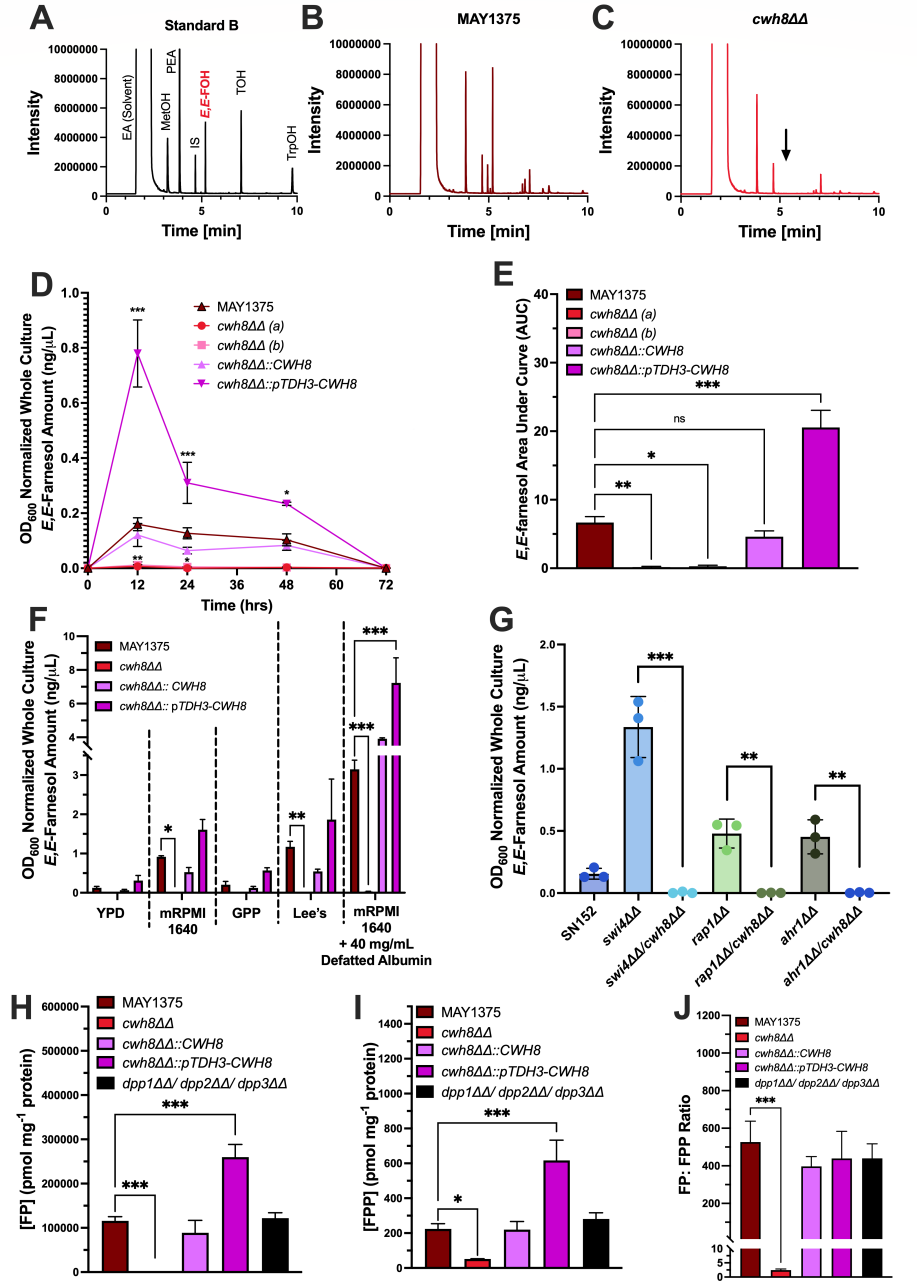
Cwh8 is responsible for much of farnesol biosynthesis in *C. albicans*. Representative gas chromatographs of ethyl acetate extracts of a known standard (13) (**A**) MAY1375 (**B**), and the *cwh8ΔΔ* mutant (**C**) grown in mRPMI 1640 at 24 hours post-inoculation showing the absence of farnesol-related peaks for *cwh8ΔΔ*. (**D**) Farnesol accumulation assessed at 12, 24, 48, and 72 h post-inoculation at 30°C in YPD for MAY1375, two independently generated *cwh8* null mutants (*a/b*), CWH8 reconstituted (*cwh8ΔΔ::CWH8*), and constitutive overexpression strain (*cwh8*ΔΔ::p*TDH3-CWH8*). Data are the mean OD_600_ normalized whole farnesol accumulation ±SEM of three independent growth curves. (**E**) Area under the curve (AUC) analysis performed on the curves presented in (**D**) and data are the mean AUC ±SEM. (**F**) Farnesol accumulation assessed at 24 h post-inoculation at 30°C in either YPD, GPP, Lee’s, mRPMI, or mRPMI supplemented with fatty acid-free albumin. (**G**) Farnesol accumulation assessed at 24 h post-inoculation at 30°C in YPD for SN152+, *swi4ΔΔ, swi4ΔΔ/cwh8ΔΔ, rap1ΔΔ, rap1ΔΔ/cwh8ΔΔ*, *ahr1ΔΔ,* and *ahr1ΔΔ/cwh8ΔΔ* null mutants. Data presented are the mean OD_600_ normalized whole farnesol accumulation ±SD. (**H**) Cellular farnesyl phosphate (FP) assessed at 24 h post-inoculation at 30°C in mRPMI-1640 by LC-MS/MS. (**I**) Cellular farnesyl pyrophosphate (FPP) assessed at 24 h post-inoculation at 30°C in mRPMI-1640 by LC-MS/MS. (**J**) Cellular FP/FPP ratios. Data presented are the mean analyte concentration (pmol) normalized to protein (mg) ±SD. Differences between groups were determined by one-way ANOVA with Dunnett’s multiple comparisons test. Differences were considered significant at *P* < 0.05 (*P < 0.05, **P < 0.01, and ***P < 0.001).

Does *Ca*Cwh8 possess activity against FPP in *C. albicans*? We wondered because FPP has only three isoprene units while *CWH8* is characterized as encoding a putative Dol-PP phosphatase, requiring substrates of 15–18 isoprene units. Also, Janik et al. (33) showed that *cwh8ΔΔ* mutants of *C. albicans* lacked Dol-PP phosphatase activity (33). We confirmed that Cwh8 contained the consensus lipid pyrophosphate catalytic domain (41). Indeed, Dpp1, Dpp2, and Dpp3 also possessed this conserved domain (Fig. 2E). We then generated two independent *cwh8ΔΔ* deletion mutants and examined their farnesol production. Dramatically, GC-FID chromatograms for whole cultures of *cwh8ΔΔ* grown in mRPMI 1640 (Fig. 3C) or YPD (Fig. S1) showed the complete disappearance of *E,E*-farnesol (RT = 5.3 min) (Fig. 3C). Triplicate 72 hour experiments in YPD showed that both independent *cwh8ΔΔ* mutants produced less than 0.1% as much farnesol as their MAY1375 parent (Fig. 3D and E). As expected, the reconstituted mutant with wild-type *CWH8* restored farnesol accumulation (Fig. 3D and E; Fig. S2). Furthermore, a null mutant containing *CWH8* driven by a constitutive promoter (p*TDH3*) exhibited a 23-fold increase in expression (see Fig. 6B) and produced ca. threefold more farnesol (Fig. 3D and E; Fig. S2), indicating that *CWH8* overexpression increased farnesol accumulation. The higher levels of farnesol in the p*TDH3-CWH8* overexpression strain were most dramatic (fivefold) at the 12 hour time point (Fig. 3D), but they were followed by a normal wild-type farnesol decay profile (Fig. 3D). The combination of these observations suggests that Cwh8 is involved in farnesol synthesis rather than farnesol decay and the mechanism for farnesol removal/decay is independent of Cwh8.

These effects of *CWH8* on farnesol production were not media-dependent; no farnesol was produced by the *cwh8ΔΔ* cells in either YPD (Fig. 3D and E; Fig. S1 and S2) or four defined growth media: GPP (42), Lee’s (43), mRPMI (13), or mRPMI +fatty acid-free albumin (44) (Fig. 3F). We previously observed that the addition of fatty acid-free albumin caused a fivefold increase in farnesol accumulation by *C. albicans* (44). Note that fatty acid-free albumin caused a threefold increase in farnesol produced by the MAY1375 parent, but none for the *cwh8ΔΔ* mutant (Fig. 3F).

To confirm the importance of *CWH8*, we next generated a series of *cwh8ΔΔ* deletions in mutants known to have higher farnesol production levels. The transcription regulator mutants *swi4ΔΔ*, *rap1ΔΔ*, and *ahr1ΔΔ* (37) were previously shown to have increased farnesol accumulation of 9-, 4.5-, and 3.5-fold, respectively (38). When repeated for this paper (Fig. 3G), *swi4ΔΔ*, *rap1ΔΔ*, and *ahr1ΔΔ* had 8.6-, 3.1-, and 2.9-fold higher farnesol accumulation at 24 h, but in each case, the double mutant with *cwh8ΔΔ* resulted in the complete loss of farnesol production (Fig. 3G; Fig. S3). Together, these findings indicate that CaCwh8 has FPP phosphatase activity and is essential for most of the farnesol production in *C. albicans*. Additionally, these findings renew the question of whether the conversion of FPP to farnesol is a one-step or two-step process (Fig. 1A).

### Cwh8 is necessary for FPP to FP conversion in *C. albicans*

Actual measurements for cellular FP and FPP concentrations are scarce in the literature, especially for fungi. This deficiency is likely caused by the detergent-like properties of both molecules, which make them difficult to separate via anionic or hydrophobic based methods. To overcome these difficulties, we have developed an LC/MS method for the simultaneous measurement of cellular FP (Fig. 3H), FPP (Fig. 3I), and the FP/FPP ratio (Fig. 3J). This method is described in detail in the supplemental Material and Methods (Text S1). The results were dramatic, in that the FP pool size (Fig. 3H) was ca. 400 times larger than the FPP pool (Fig. 3I) for the *C*. *albicans* parent MAY1375, the *cwh8ΔΔ::CWH8* reconstituted strain, the *cwh8ΔΔ*::p*TDH3-CWH8* overexpressing strain; and the *dpp1ΔΔ/dpp2ΔΔ/dpp3ΔΔ* triple mutant (Fig. 3J). In contrast, the *cwh8ΔΔ* mutant had an FP/FPP ratio of ca. 2.4 (Fig. 3J). These results are consistent with Cwh8 converting FPP to FP, in that the *cwh8ΔΔ* mutants had ca. 950-fold less FP than their parent (Fig. 3H) but only 4.5-fold less FPP (Fig. 3I). Thus, the enzymatic conversion of FPP to FP by Cwh8 is essential for farnesol production (Fig. 3), but it may not be the rate-limiting step in farnesol biosynthesis; other as yet unidentified enzymes, possibly including Dpp1-3 and/or Cwh8 itself, operating at lower efficiency, convert FP to farnesol. In support of this model, the *dpp1ΔΔ/dpp2ΔΔ/dpp3ΔΔ* triple mutant has a 14% increase in FP compared to its MAY1375 parent (Fig. 3H).

### Cwh8 is essential for cell wall homeostasis: Growth-related phenomena

**Fig 4:**
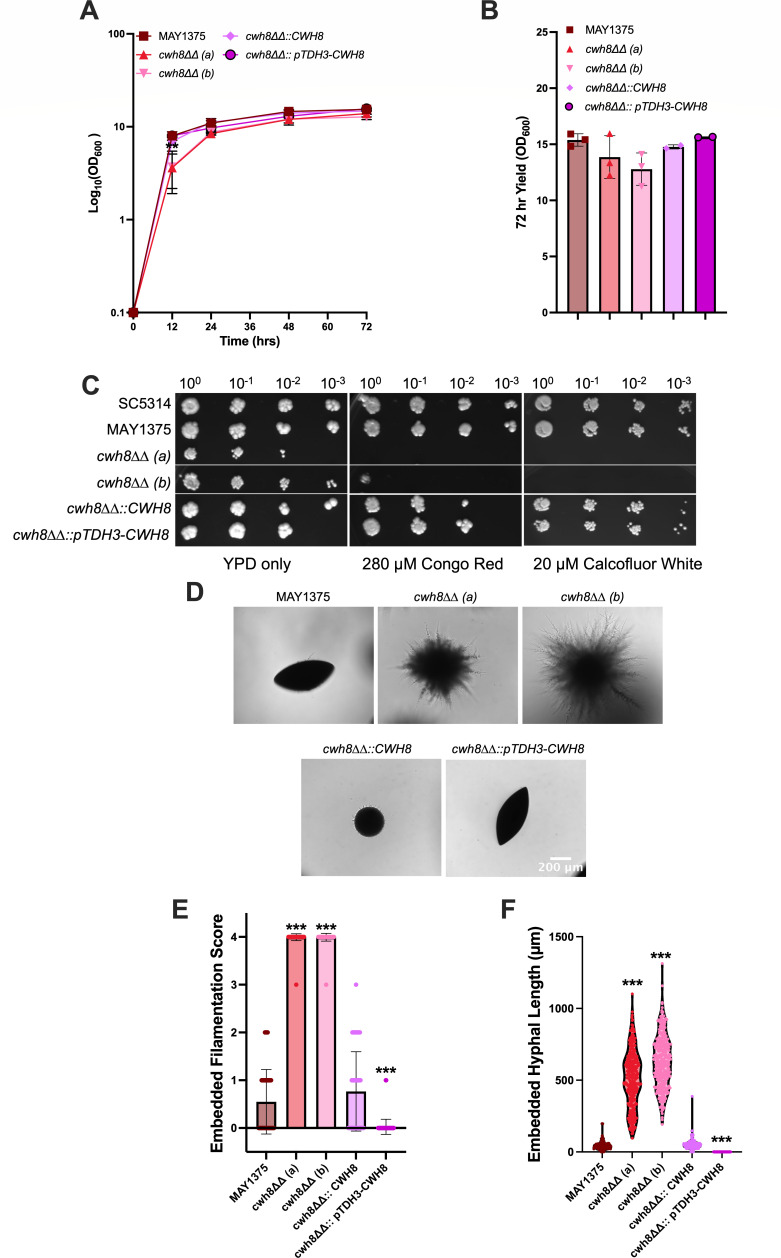
*cwh8ΔΔ* mutants exhibit a growth delay, decreased cell yields, and decreased resistance to cell wall stress, yet they still demonstrate increased filamentation in embedded conditions. (**A**) Growth curve and (**B**) total yield after 72 h in YPD medium (OD_600_) of MAY1375, *cwh8ΔΔ*., *cwh8ΔΔ.::CWH8* and *cwh8ΔΔ.::pTDH3-CWH8*. Data represent the mean growth value (OD_600_) ±SD (*n* = 3). (**C**) The susceptibility of the mutants to cell wall (20 µM Calcofluor White and 280 µM Congo Red) stressors, as determined by serial dilution growth tests. (**D**) Cells of MAY1375, *cwh8ΔΔ*., *cwh8ΔΔ.::CWH8,* and *cwh8ΔΔ.::pTDH3-CWH8* were submersed in molten YPS agar and grown for 5 days at 25°C. Bar, 200 µm. Images are representative of three independent experiments. (**E**) Embedded filamentation score. Data presented are the mean filamentation score ±SD of >150 colonies across three independent experiments using the criteria from Azadmanesh et al. 2017 (48). (**F**) Hyphal length of embedded colonies. Data are the mean hyphal length ±SD for at least 100 hyphae across three independent experiments. Differences between groups were determined by one-way ANOVA with Dunnett’s multiple comparisons test. Differences were considered significant at *P* < 0.05 (*P < 0.05, **P < 0.01, and ***P < 0.001).

 By serving as carrier lipids, dolichols have a variety of critical cellular functions (45) including the synthesis of glycoproteins, GPI-anchored proteins, and cell wall mannans (32–34); cellular defense against oxidative stress (46); and contributing to the proper formation and fluidity of cell membranes (47). Given the diversity of functions requiring dolichol or Dol-P, we next examined the growth characteristics and stress tolerance profiles exhibited by *cwh8ΔΔ* null mutants. Growth of the *cwh8ΔΔ* mutants in YPD was delayed or slowed (note the 2.2-fold lower cell density at 12 h (Fig. 4A), and their final yields (OD_600_) at 72 h were reduced by an average of 13.5% when compared to their MAY1375 parent (Fig. 4B). We next performed serial dilution growth tests on YPD supplemented with a series of stress agents. The two independent *cwh8ΔΔ* strains exhibited slight growth defects on YPD only (Fig. 4C), but no further differences on plates supplemented with 5 mM H_2_O_2_ or 1.5 M sorbitol, thus revealing no changes in oxidative or osmotic stress tolerance (data not shown). In contrast, *cwh8ΔΔ* mutants exhibited greatly reduced growth in the presence of 280 µM congo red or 20 µM calcofluor white (Fig. 4C), supporting the conclusion that Cwh8 is involved in cell wall homeostasis (33). At present, we cannot distinguish whether these growth/stress defects are due to the absence of farnesol and/or reduced levels of dolichyl-P.

### Cwh8-mediated farnesol production influences embedded filamentation

Farnesol is a well-documented negative regulator of filamentation in *C. albicans* (12, 14, 16), and thus we hypothesized that endogenous farnesol accumulation as mediated by Cwh8 may modulate filamentation. Consistent with prior reports (33), the *cwh8ΔΔ* mutants were significantly delayed in liquid filamentation assays (Fig. S4) likely due to their aberrant cell walls (33). Moreover, we observed no discernible differences in the capacity of the *pTDH3-CWH8* overexpression strain to filament under liquid conditions (Fig. S4). In both cases, endogenous farnesol could be diluted by fresh medium during the dilution steps, meaning that causal interpretations would indeed be difficult. Therefore, to establish whether Cwh8-mediated farnesol production is a determinant of filamentation, we investigated its effects in embedded assays where local accumulation of farnesol may exert greater significance and the time needed for filamentation could prove less critical than in liquid media with shaking. Under embedded conditions, the two independent *cwh8ΔΔ* mutants exhibited robust filamentation (Fig. 4D and E), while the overexpression strain displayed an almost complete absence of filamentation after 5 days in YP sucrose agar at room temperature (Fig. 4D and E). Furthermore, *cwh8ΔΔ* mutants exhibited significantly increased filament frequency (Fig. 4E) and length (Fig. 4F) than their MAY1375 parent or the *CWH8* reconstituted strain (Fig. 4F). Collectively, these observations are consistent with Cwh8 regulation of farnesol in ways that affect the biology of *C. albicans*.

### *CWH8*-mediated farnesol production influences the expression of pathogenicity-related genes and multidrug efflux pumps

**Fig 5:**
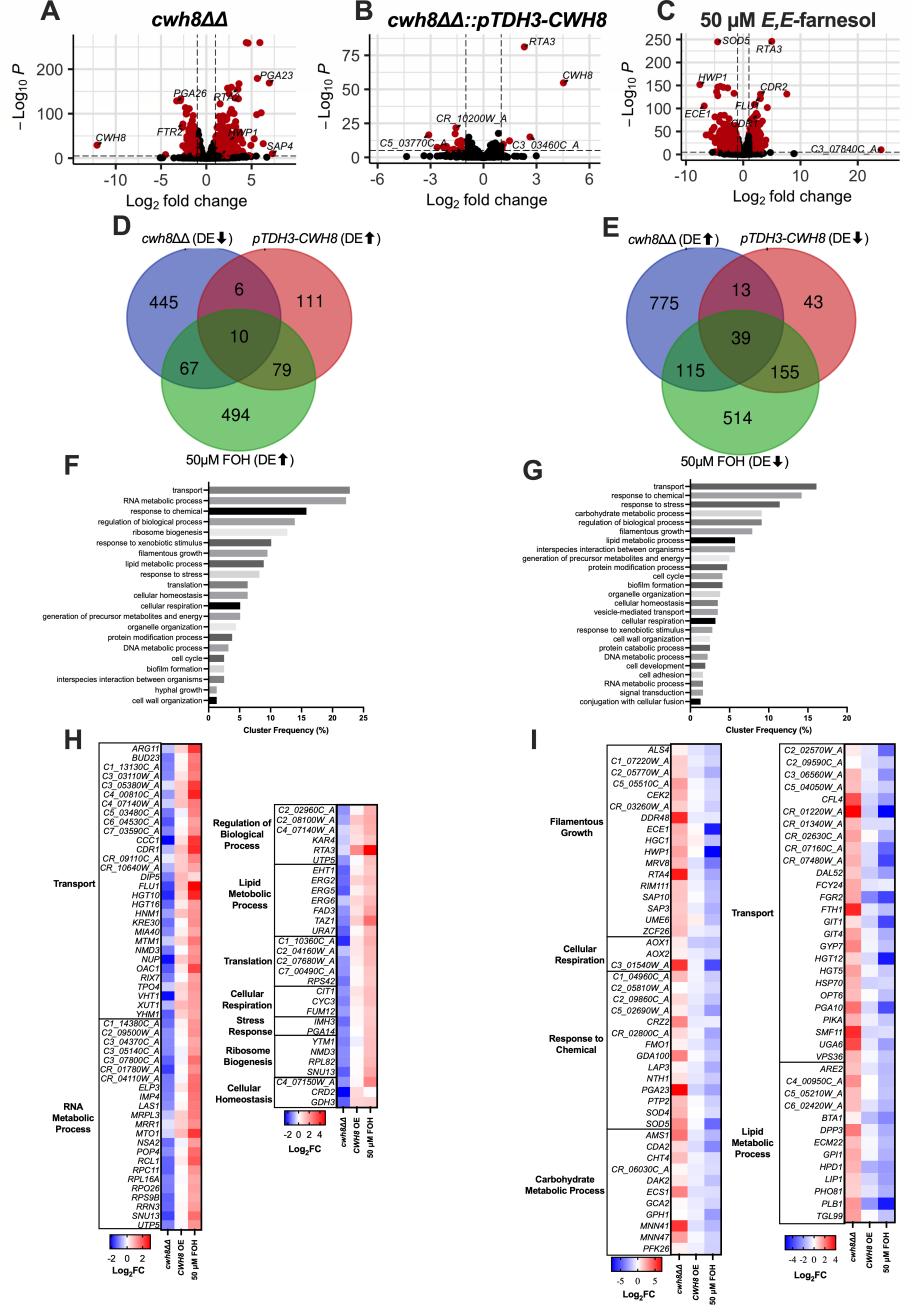
*CWH8* overexpression is sufficient to copy the effect of exogenous farnesol exposure in upregulating multidrug resistance genes and downregulating pathogenesis-related genes. Four biological replicates of MAY1375, *cwh8∆∆*, and *cwh8∆∆::pTDH3-CWH8* were prepared in mRPMI 1640 and diluted to an OD600 of 0.1 in fresh mRPMI 1640 and harvested after 6 h at 30°C. Additionally, MAY1375 cultures were prepared with and without 50 µM *E,E*-farnesol addition at time zero. Differential gene expression was analyzed using thresholds of an adjusted *P*-value < 0.05 and a log2 fold change (log2FC) cutoff of ≥1.0 for the *cwh8∆∆* mutant and farnesol-treated cells, and ≥0.5 for the *cwh8∆∆::pTDH3-CWH8* strain. Volcano plots illustrate differentially expressed (DE) genes in (**A**) *cwh8∆∆*; (**B**) *cwh8∆∆::pTDH3-CWH8*; and (**C**) 50 µM FOH-treated cells. Gene overlap analyses display (**D**) DE genes upregulated by either FOH treatment or *cwh8∆∆::pTDH3-CWH8* that are downregulated in *cwh8∆∆* and (**E**) DE genes downregulated by either FOH or *cwh8∆∆::pTDH3-CWH8* that are upregulated in *cwh8∆∆*. These analyses include genes overlapping between any two of the three conditions. GO Slim term analyses display the cluster frequency of the overlapping (**F**) DE genes upregulated by either FOH treatment or *cwh8∆∆::pTDH3-CWH8* that are downregulated in *cwh8∆∆*, and (**G**) DE genes downregulated by either FOH or *cwh8∆∆::pTDH3-CWH8* that are upregulated in *cwh8∆∆*. Heatmaps present overlapping genes categorized by GO Slim terms (CGD): (**H**) genes upregulated by FOH and *cwh8∆∆::pTDH3-CWH8* but downregulated in *cwh8∆∆*, and (**I**) genes downregulated by FOH and *cwh8∆∆::pTDH3-CWH8* but upregulated in *cwh8∆∆*. Heatmap values represent log2FC in gene expression relative to untreated MAY1375, with blue indicating downregulation and red indicating upregulation.

 We hypothesized that the altered levels of farnesol production, stemming from either the loss or overexpression of *CWH8*, would lead to changes in the expression profiles of farnesol-responsive genes. To investigate this hypothesis, we performed comprehensive RNA-seq analysis of the *cwh8ΔΔ* and *cwh8ΔΔ::pTDH3-CWH8* strains, as well as MAY1375 grown with 50 µM E,E-farnesol, all compared with MAY1375 without farnesol (Fig. 5). The rationale of choosing 30°C was for two reasons: to coincide with all the other growth and farnesol production reported in Fig. 1 to 4 and to avoid the complexity of cells carrying out GTF or mycelial growth. From a total of 6147 genes, we identified 1470, 456, and 1473 differentially expressed genes (DEGs) (Fig. 5A through C; Table S4). We then arranged these DEGs in two Venn diagrams, comparing the downregulated genes of *cwh8ΔΔ* with those upregulated by either *pTDH3-CWH8* or farnesol exposure (Fig. 5D) and vice versa (Fig. 5E) and found 162 and 322 shared genes, respectively (Table S5). The diversity and sheer number of genes influenced by farnesol exposure supports the theme developed earlier (27) that farnesol has basic roles in *C. albicans* physiology and pathogenicity beyond just acting as a quorum sensing molecule.

To further characterize these overlapping gene sets, we performed GO Slim analysis (Fig. 5F and G; Table S6). Of the shared genes, 184 (37 + 147) were annotated with unknown biological processes. The most enriched biological processes are shown in the heatmaps for the differentially downregulated genes of *cwh8ΔΔ* with those upregulated by either *pTDH3-CWH8* or farnesol exposure (Fig. 5H) and vice versa (Fig. 5I). This analysis reveals a broad spectrum of biological processes influenced by each condition. We will focus on the genes involved in transport, filamentous growth, and lipid metabolism (Fig. 5H and I).

### Transport-related genes

Consistent with prior reports (49–52), exogenous farnesol exposure upregulated the expression of key drug efflux regulators and genes (Fig. 5H). Specifically, the expressions of *CDR1, MRR1, FLU1,* and *RTA3* were upregulated in the presence of exogenous farnesol (Fig. 5H). Moreover, we found that the *CWH8* overexpression exhibited a similar upregulation of these drug efflux genes consistent with its increased farnesol accumulation. In contrast, the *cwh8ΔΔ* mutant exhibited a distinct downregulation of these drug efflux genes (Fig. 5H).

 Exogenous farnesol exposure, and to a lesser extent the *pTDH3-CWH8* strain, also upregulated the expression of genes involved in the transport of amino acids (*ARG11 and DIP5*), sugars (*HGT10 and HGT16*), citric acid cycle intermediates (*OAC1 and YHM1),* metal ions (*CCC1 and MTM1*), and small molecules and vitamins (*TP04, VHT1, and XUT1*) that were all downregulated in the *cwh8ΔΔ* mutant (Fig. 5H). Conversely, exogenous farnesol exposure and the *pTDH3-CWH8* strain downregulated other genes involved in sugar transport (*HGT5, HGT12, GIT1, and GIT4*), metal ion transport (*FTH1, SMF11, and PGA10*), nitrogen source transport (*OPT6 and DAL52*), protein transport (*HSP70*), and vacuolar trafficking (*VSP36 and GYP7*) that were all upregulated in the *cwh8ΔΔ* mutant (Fig. 5I).

### Filamentous growth-related genes

Consistent with its lack of farnesol production (Fig. 3) and increased embedded filamentation (Fig. 4), *cwh8ΔΔ* cells had significantly increased expression of hyphal-specific genes (*HGC1* and *HWP1*), virulence genes (*ECE1, MRV8, and HSP70),* lipases (*LIP1* and *PLB1*), signaling proteins (*CEK2 and ZCF26*), morphogenesis regulators (*UME6* and *RIM111*), and proteases (*SAP3* and *SAP10*) (Fig. 5I). In contrast, farnesol-treated cells exhibited markedly reduced expression of these genes, aligning with its role as a repressor of filamentation (12, 16, 50). The *pTDH3-CWH8* strain demonstrated a similar pattern of downregulation, albeit to a lesser extent.

### Lipid metabolism-related genes

Farnesol is a hydrophobic molecule with a maximum water solubility of ca. 1 mM (28). Thus, added farnesol is necessarily membrane-bound (31, 53) such that 6–8 washes are needed to remove all the cell-bound farnesol (44). We are interested in the structural adaptations cells make to compensate for higher levels of farnesol in their cellular membranes. Farnesol exposure increased expression of ergosterol biosynthesis genes (*ERG2, ERG5, and ERG6*), lipid metabolism enzymes (*FAD3, TAZ1, and EHT1*), and lipid regulators (*RTA3 and KAR4*), all of which were similarly downregulated in the *cwh8ΔΔ* mutant (Fig. 5H). In contrast, the *cwh8ΔΔ* mutant upregulated genes involved in phosphate metabolism (*PHO81*), sterol metabolism (*ARE2, ECM22*), and various lipid metabolism enzymes (*BTA1, DPP3, GPI1, HPD1, LIP1, PLB1, and TGL99*), all of which were downregulated upon farnesol treatment (Fig. 5I).

### Differences in Cwh8 specificity explain differences in farnesol accumulation across species

**Fig 6:**
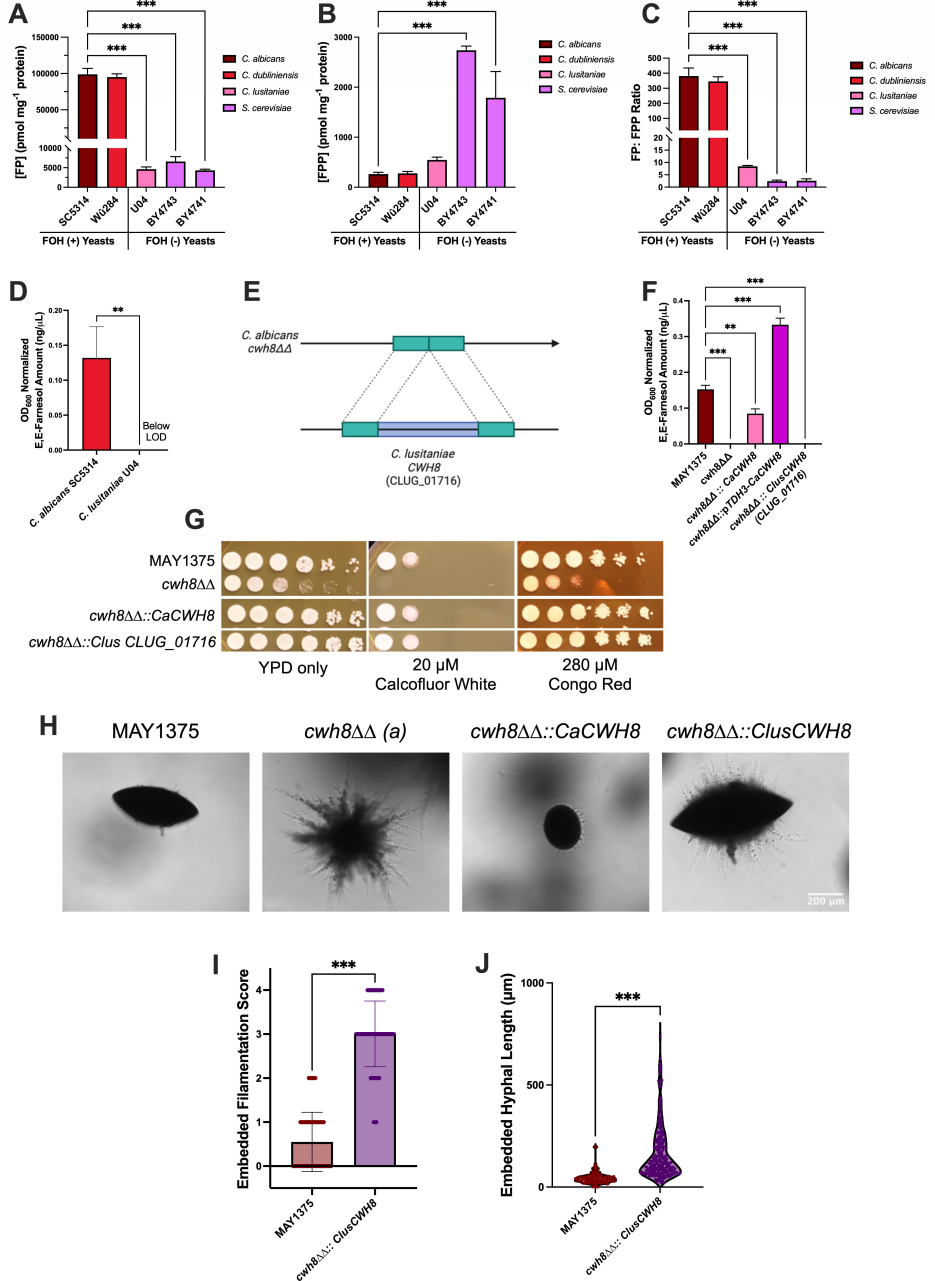
Differences in Cwh8 explain differences in farnesol accumulation across yeasts. (**A**) Cellular farnesyl phosphate (FP) and (**B**) farnesyl pyrophosphate (FPP) assessed at 24 h post-inoculation at 30°C in mRPMI-1640 by LC-MS/MS. (**C**) Cellular FP/FPP ratios. (**D**) Farnesol accumulation assessed at 24 h post-inoculation at 30°C in YPD for *C. albicans* SC5314 and *C. lusitaniae* U04. LOD= limit of detection. Data presented are the mean OD_600_ normalized whole farnesol accumulation ±SD. (**E**) Strategy for complementation of *C. albicans cwh8ΔΔ* with *C. lusitaniae* (CLUG_01716). (**F**) Farnesol accumulation assessed at 24 h post-inoculation at 30°C in YPD for MAY1375, *cwh8ΔΔ, cwh8ΔΔ.::CaCWH8, cwh8ΔΔ.::pTDH3-CaCWH8, and cwh8ΔΔ:: C. lusitaniae* (CLUG_01716). (**G**) Serial dilution growth tests on YPD agar media with the addition of either calcofluor white (20 µM) or Congo red (280 µM). (**H**) Cells of MAY1375, *cwh8ΔΔ, cwh8ΔΔ.::CaCWH8, and cwh8ΔΔ :: C. lusitaniae* (CLUG_01716) were submersed in molten YPS agar and grown for 5 days at 25°C. Bar, 200 µm. Images are representative of three independent experiments. (**I**) Embedded filamentation score. Data presented are the mean filamentation score ±SD of >150 colonies across three independent experiments using the criteria from Azadmanesh et al. 2017 (48). (**J**) Hyphal length of embedded colonies. Data are the mean hyphal length ±SD for at least 100 hyphae across three independent experiments. Note: MAY1375 embedded filamentation data are the same as that shown in Fig. 4 and are reshown to enable convenient comparison. Differences between groups were determined by one-way ANOVA with Dunnett’s multiple comparisons test. Differences were considered significant at *P* < 0.05 (*P < 0.05, **P < 0.01, and ***P < 0.001).

Given that the FP/FPP ratio for MAY1375 was dramatically skewed toward FP (Fig. 3H), we wondered whether this large FP pool was common to other yeasts. To address this question, we quantified FP (Fig. 6A) and FPP (Fig. 6B) in two farnesol-secreting yeast species *C. albicans* (SC5314) and *C. dubliniensis* (Wü284) and two farnesol non-secreting species *Clavispora lusitaniae* (U04) and *S. cerevisiae* (BY4741 and BY4743). The FP pool was approximately 400 times larger than the FPP pool in *C. albicans* and *C. dubliniensis* (Fig. 6C). In contrast, the farnesol non-secreting yeasts exhibited much lower FP/FPP ratios—2.4 for the *S. cerevisiae* strains and 8.45 for *C. lusitaniae* (Fig. 6C). Thus, there is a clear distinction between these groups, with known farnesol secretors exhibiting FP/FPP ratios roughly 160-fold higher than nonsecretors. The large differences in FP/FPP ratios among yeast species led us to hypothesize that the Cwh8 protein may be functionally divergent in farnesol-secreting yeasts. To test this hypothesis, we examined a *cwh8Δ* mutant of *S. cerevisiae* (cax4Δ) and found that compared with its parent, its FP pool decreased by approximately 29% (Fig. S5A), a much smaller reduction than that observed in the *C. albicans cwh8ΔΔ* mutant (Fig. 3H).

To further investigate whether Cwh8 function is conserved across yeast species, we expressed a *CWH8* homolog (CLUG_01716) from *C. lusitaniae*, a species that does not secrete detectable farnesol under normal conditions (Fig. 6D), at the native *CWH8* locus in *C. albicans* (Fig. 6E). Significantly, complementation of the *C. albicans cwh8ΔΔ* mutant with the *C. lusitaniae CWH8* homolog restored tolerance to Calcofluor White and Congo Red (Fig. 6G), but failed to restore farnesol production (Fig. 6F). Furthermore, consistent with its lack of farnesol production, the *cwh8ΔΔ::CLUG_01716* strain exhibited increased filamentation on YPS agar at 25°C (Fig. 6H and I) and formed longer filaments than its MAY1475 parent (Fig. 6J). Collectively, these results indicate that Cwh8’s role in maintaining cell wall integrity is conserved between species, but its participation in farnesol biosynthesis is not. Thus, *C. albicans* may have evolved to secrete farnesol based on Cwh8 differences in substrate specificity or feedback inhibition, or on interactions with species-specific cofactors.

### Absence of a trailing end phenotype in the *cwh8 ∆∆* mutant treated with fluconazole

**Fig 7:**
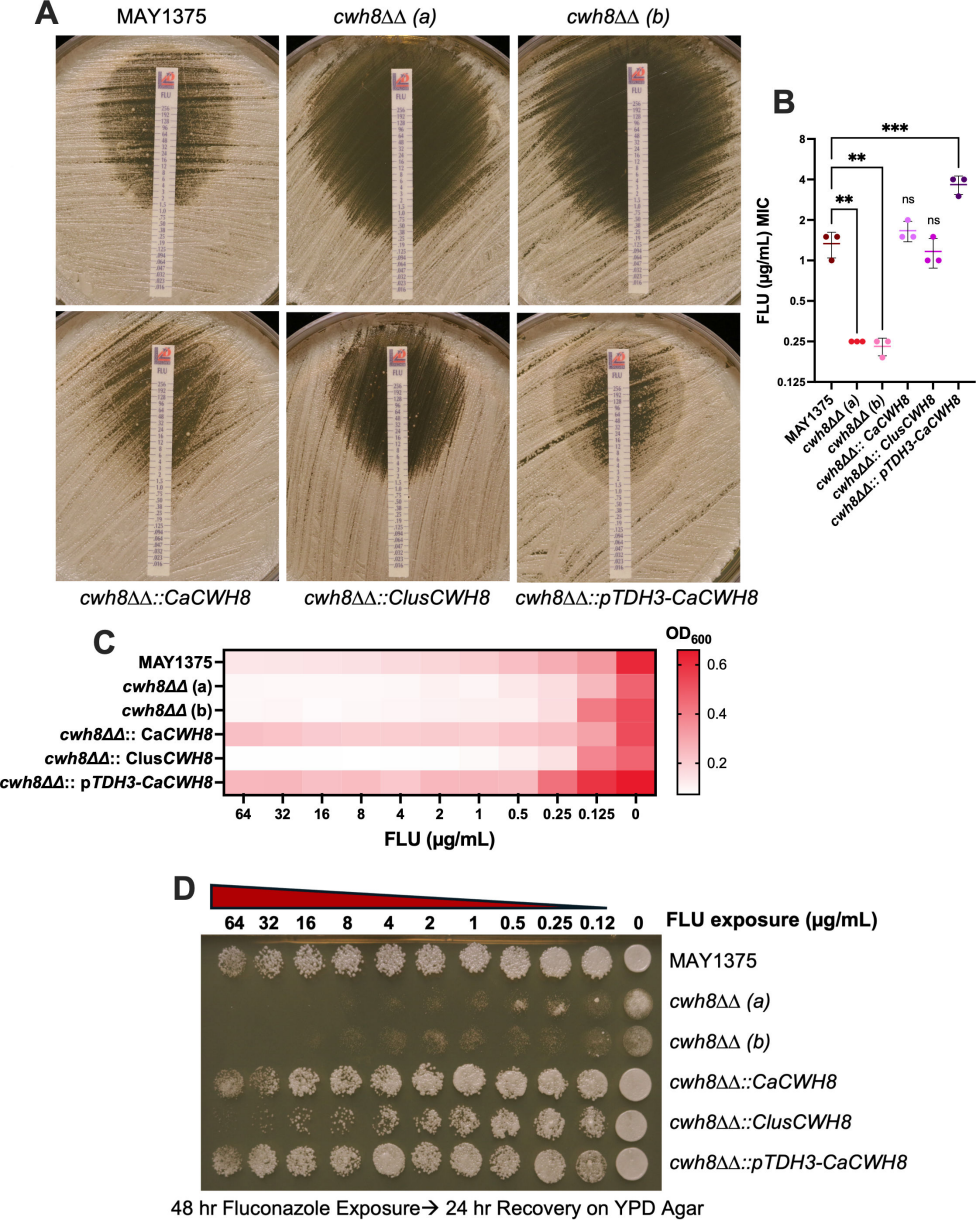
Absence of a trailing end phenotype in the *cwh8∆∆* mutant treated with fluconazole. Susceptibility of MAY1375, *cwh8∆∆*, *cwh8∆∆::CaCWH8*, *cwh8∆∆::ClusCWH8,* and *cwh8∆∆::pTDH3-CaCWH8* strains to fluconazole was assessed using the following assays: (**A**) Etest on YPD plates incubated for 48 hours at 30°C, with mean minimum inhibitory concentration (MIC) values ± SD shown in (**B**); and (**C**) CLSI broth microdilution assay in RPMI 1640. Broth microdilution data represent the mean OD_600_ after 48 hours (*n* = 3). (**D**) Recovery of cells exposed to fluconazole for 48 hours, plated on YPD agar and incubated for 24 hours. Images are representative of three biological replicates. Differences between groups were determined by one-way ANOVA with Dunnett’s multiple comparisons test. Differences were considered significant at *P* < 0.05 (*P < 0.05, **P < 0.01, and ***P < 0.001).

Given the upregulation of genes encoding multidrug resistant (MDR) efflux pumps by farnesol (49, 50) (Fig. 5H), we hypothesized that endogenous farnesol may modulate resistance to antifungals. SD medium supplemented with fluconazole or terbinafine showed that *cwh8ΔΔ* was slightly more sensitive to both antibiotics (Fig. S6). When tested on YPD agar with Etest strips of fluconazole (Fig. 7A), the MIC values averaged 0.25 µg/mL for the *cwh8ΔΔ* strains versus 1.33 µg/mL for the parent and 1.66 µg/mL for the *CaCWH8* reconstituted strain (Fig. 7B). Notably, the *CaCWH8* overexpression strain increased fluconazole resistance, raising the MIC to 3.66 µg/mL. Strikingly, both *cwh8∆∆* mutants lacked the characteristic trailing endpoint phenotype, where *Candida* spp. exhibit reduced yet persistent growth at fluconazole concentrations above the MIC (54). The agar near higher fluconazole concentrations remained clear for *cwh8∆∆*, lacking late-developing colonies (Fig. 7A). This absence was further confirmed in broth microdilution assays with RPMI 1640 medium containing increasing fluconazole (Fig. 7C), where parental, *CaCWH8* reconstituted, and *CaCWH8* overexpression strains showed typical late growth, while *cwh8∆∆* cells were highly sensitive, with limited growth above 0.25 µg/mL (Fig. 7C). The lack of growth within the Etest clearing zones suggested that fluconazole might be fungicidal to *cwh8∆∆* mutants (Fig. 7A), consistent with the complete loss of trailing endpoint growth in broth assays (Fig. 7C). To test this hypothesis, 48 hour fluconazole-exposed cells from the microdilution assays were plated on YPD to assess recovery (Fig. 7D). Viability dramatically decreased with increasing fluconazole concentrations in *cwh8∆∆* cells, but not in parental, *CaCWH8* reconstituted, or *CaCWH8* overexpression strains. Similarly, the *cwh8∆∆::CLUG_01716* mutant, which cannot produce farnesol (Fig. 6F), showed reduced trailing growth and decreased viability at high fluconazole levels, albeit to a lesser degree (Fig. 7A, C and D). One explanation for these differences invokes the finding of Liu et al. (49) that farnesol can activate the multidrug efflux pump Cdr1, thus lowering the cytoplasmic fluconazole levels.

## DISCUSSION

 How *C. albicans* synthesizes farnesol has been an outstanding question for over 20 years (26–28, 35). Here, we have shown that Cwh8 is essential for farnesol synthesis in *C. albicans*. Independently generated *cwh8ΔΔ* mutants produced no detectable farnesol in a wide variety of growth media, even in strains that would otherwise produce exceptionally high levels of farnesol. In *S. cerevisiae*, *CWH8* encodes a dolichyl pyrophosphate phosphatase with a luminally oriented active site located in the endoplasmic reticulum (32). Cwh8 catalyzes the removal of phosphate from Dol-PP by cleavage of the phosphodiester bond to form stoichiometric amounts of Pi and Dol-P (Fig. 1A), but it was also capable of dephosphorylating Dol-P, albeit at a markedly slower rate (32). Thus, assuming that Cwh8 accomplishes the FPP to FP conversion (Fig. 3), our data are consistent with Dpp1, Dpp2, Dpp3, and/or an as yet unidentified phosphatase acting as the second part of a two-step process via the dephosphorylation of FP. Having established the role of Cwh8 in farnesol biosynthesis, we will now discuss more focused questions surrounding its function.

### Trailing endpoint phenomenon in *C. albicans* fluconazole susceptibility testing

 Trailing endpoint growth is a phenomenon where *Candida* spp. continue to grow at low levels, even at concentrations of antifungal drugs higher than the minimum inhibitory concentration (MIC), thus making it difficult to interpret the results of antifungal drug susceptibility tests (54). Soon after our first publication on farnesol (12), Ted White (University of Missouri-Kansas City) suggested to us that trailing endpoint growth and farnesol might be mechanistically related. What we needed was an otherwise normal mutant that did not make any farnesol. *cwh8ΔΔ* does not produce detectable farnesol (Fig. 3) and also does not exhibit trailing growth (Fig. 7). These observations suggest the following as a possible five-part scenario: fluconazole is normally fungistatic for *C. albicans*. It acts to inhibit ergosterol biosynthesis; subinhibitory levels of azole antibiotics increase the secretion of farnesol by 20-40 fold (55); fluconazole turns on farnesol synthesis by increasing the expression of *CWH8*. Rogers and Barker (56) showed that fluconazole-resistant mutants of *C. albicans* expressed 3.6-fold more *CWH8* (56), while *CWH8* expression was increased 3.2-fold by 10 µM itraconazole (57) and 2.6-fold by ketoconazole (58). However, it was not upregulated by amphotericin B, caspofungin, or 5-flucytosine (58). In *C*. *albicans*, the accumulated farnesol upregulates multidrug efflux pumps, such as *CDR1* (49, 50, 52, 59, 60) (Fig. 5H). Note that each of the references cited used physiologically relevant farnesol concentrations, ranging from 4 to 50 µM. The fluconazole-resistant mutants mentioned previously also expressed 3.6-fold more *CDR1* (56), and *CDR1* was upregulated 5.9-fold by ketoconazole (58) but not by amphotericin B, caspofungin, or 5-flucytosine (58). The intracellular levels of fluconazole are thus lowered, permitting growth to resume.

 Sharma and Prasad (61) provide an alternative view by which 100 µM farnesol inhibits the efflux of rhodamine 6G by Cdr1 and Cdr2 (61), thus supporting the benefits of using azole antibiotics and efflux pump inhibitors in combination (61). However, these conclusions were based on cells prepared under very different conditions. Typically, they used non-growing cells with *CDR1* already overexpressed, suspended in PBS without glucose. Moreover, the cells had been deenergized by prior treatment with 5 mM 2-deoxyglucose and 5 mM dinitrophenol. Thus, these cells are very different physiologically from actively growing yeasts. A remarkably similar scenario has played out in *C. auris* where different concentrations of exogenous farnesol led to very different conclusions. Jakab et al. (51) used 75 µM farnesol and concluded that the added farnesol led to increased expressions of *CDR1*, *CDR2*, and *MDR1* 2 hours later. In contrast, Dekkerová et al. (62) used 200 µM farnesol and concluded that the added farnesol blocked the Cdr1 efflux pump, acted synergistically with fluconazole, and caused excess fluconazole and rhodamine 6G to accumulate intracellularly. Cao et al. (52) evaluated multiple farnesol concentrations and concluded that 40 µM was the highest concentration that suppressed biofilm formation in *C. albicans* without causing other cellular damage. We agree that a concentration above 40–50 µM farnesol should no longer be considered physiologically relevant.

 Another potential model is that the ability to secrete farnesol may contribute to increased fluconazole resistance by alleviating intracellular accumulation of toxic intermediates in the ergosterol biosynthesis pathway, such as farnesyl pyrophosphate (FPP) or isopentenyl pyrophosphate (IPP) (63–65). Fluconazole inhibits lanosterol 14α-demethylase, causing a buildup of upstream metabolites that can be cytotoxic if not efficiently processed or removed. A good example is the toxic sterol 14α-methylergosta-8,24 (28)-diene-3β,6α-diol that permeabilizes the plasma membrane and arrests fungal growth (66). By secreting farnesol, a downstream isoprenoid derivative of FPP, cells potentially reduce the intracellular concentrations of FPP and IPP, thereby limiting their toxic effects. This detoxification mechanism may help maintain cellular homeostasis under antifungal stress, supporting survival and tolerance to fluconazole. Consistent with this model, the *cwh8∆∆::CLUG_01716* mutant, which lacks endogenous farnesol production (Fig. 6F), exhibits reduced fluconazole trailing growth and decreased viability upon fluconazole exposure, though to a lesser extent than the *cwh8∆∆* deletion mutant (Fig. 7C and D). This partial phenotype suggests that farnesol secretion is a factor in detoxifying intermediates and promoting fluconazole tolerance, supporting a protective role for farnesol secretion against the toxic buildup of FPP and IPP under antifungal stress.

### Both large and small substrates

*CWH8* encodes a protein of 241 amino acids with four predicted transmembrane domains. At first, it seems surprising that the same enzyme can have substrates varying from three isoprenes (FPP) up to ca. 15–18 isoprenes (Dol-PP). The small substrates are evident in the absence of farnesol in *cwh8ΔΔ* mutants (Fig. 3), while the large substrates for those same mutants are evident in their sensitivity to Congo Red and Calcofluor White (Fig. 4C) as well as the extensive characterization done by Janik et al. (33). Precedent for this duality in substrate size comes from studies on the bacterium *Escherichia coli* by Wang et al. (67). They studied intracellular FPP accumulation as a stress, which was overcome by the expression of two endogenous phosphatases that hydrolyzed FPP as their “moonlight” activity to reduce FPP toxicity. They screened nine phosphatase genes upregulated by FPP accumulation and showed that two integral membrane phosphatases (YbjG and PgpB) exhibited FPP hydrolysis with increased farnesol production (67). Importantly, YbjG is an undecaprenyl (C_55_) diphosphate (UPP) phosphatase that catalyzes the dephosphorylation of UPP to UP, an eleven isoprene carrier lipid, which is recycled during peptidoglycan synthesis. Having an enzyme with an active site able to accommodate either FPP, UPP, or Dol-PP seems implausible, but Cwh8 might get around this difficulty by having the respective substrates delivered by distinctive isoprene binding scaffold proteins or merely by having the isoprene portions of their substrates remaining membrane bound throughout.

### Cwh8 and dolichyl pyrophosphate recycling

The role of Cwh8 as a dolichyl pyrophosphate phosphatase has been well studied in *C. albicans* by Janik et al. (33). They found that *cwh8ΔΔ* differed from its CAI4 parent, in that it had defective protein N-glycosylation, cell wall integrity, filamentous growth, and biofilm formation. Both the mutant and parent still made dolichols with 15–17 isoprenes, but the mutant had a total dolichol content ca. 2.2-fold higher than that of the parent (33). This increase is to be expected. Normally, the Dol-P needed for growth is formed by both *de novo* synthesis and the recycling of Dol-PP. For *cwh8ΔΔ*, with Dol-PP recycling blocked or impaired, it needs greater *de novo* synthesis of Dol-P to continue growth. However, this enhanced synthesis, coupled with the accumulation of surplus Dol-PP, means that growth of the mutant will be energetically less efficient. This prediction for *cwh8ΔΔ* was fulfilled in our studies, in that growth was delayed or slowed (Fig. 4A) and the cell yield at the stationary phase was reduced (Fig. 4B). For triplicate cultures, the cell yields were reduced by 15%, and the cell density (OD_600_) achieved at 12 h was only 3.6 as opposed to 7.9 for the parent.

The major emphasis of Janik et al. (33) was on deficiencies in protein N-glycosylation, but they also showed that *cwh8ΔΔ* was defective in cell wall integrity and filamentous growth. With regard to cell wall integrity, our results showing sensitivity toward Calcofluor White and Congo Red (Fig. 4C) confirm their observations (33). They further showed that the increased sensitivity of *cwh8ΔΔ* to cell wall perturbing agents was likely the result of changes in its cell wall composition: its chitin content was increased by 40% compared to the wild type, while its glucose- and mannose-containing polymer levels were decreased by 40% and 73%, respectively (33). For filamentation, they showed the absence of visible hyphae after 7 days at 30°C on solid 10% horse serum or Spider medium (33). We expanded on those results by showing that *cwh8ΔΔ* filamentation was reduced to only 20% and 70% in two defined liquid media, GPP with 10 mM N-acetylglucosamine (Fig. S4A) and RPMI 1640 in 5% CO_2_ (Fig. S4B), respectively, both incubated for 4 h at 37°C. These filamentation delays likely reflect altered cell wall structure resulting from insufficient Dol-P rather than from the absence of farnesol, especially since decreased farnesol usually promotes filamentation (12, 15). This idea is supported by the extensive filamentation of embedded *cwh8ΔΔ* cells at 25°C (Fig. 4D through F). Furthermore, cells overexpressing *CWH8* showed the absence of embedded filamentation (Fig. 4D through F).

### Farnesol: A regulator of *C. albicans* morphogenesis, drug resistance, and stress adaptation

Farnesol’s role as a signaling molecule in *C. albicans* is well established: exogenous farnesol upregulates multidrug resistance genes (49, 50, 52, 59, 60) and represses the yeast-hypha transition (12, 16, 50, 52). However, until now, it has remained unclear whether these transcriptional programs respond solely to extracellular farnesol or also to the cell’s own endogenous production. Our RNA-seq analysis shows that *CWH8* overexpression largely recapitulates the signature of farnesol responsive genes, albeit to a lesser level than exogenously supplied farnesol. *pTDH3-CWH8* mimics exogenous farnesol in upregulating drug‐resistance genes (Fig. 5H) and downregulating hypha-specific/virulence markers (Fig. 5I), whereas the *cwh8ΔΔ* mutant, unable to produce farnesol, shows the opposite signature downregulating resistance genes (Fig. 5H) and upregulating hypha-specific and pathogenesis-related genes (Fig. 5I).

Collectively, by modulating *CWH8* expression, we not only recapitulate but also broaden the classic transcriptional signature of farnesol signaling. By acting as a metabolic valve for endogenous farnesol, *CWH8* thereby influences drug resistance, morphogenesis, sterol homeostasis, and stress adaptation in *C. albicans*.

### Functional divergence of Cwh8 influences farnesol production in *Candida albicans*

*CWH8* encodes a dolichyl pyrophosphate phosphatase, which is broadly conserved among eukaryotes, including a mammalian homolog *DOLPP1* (68). Defects in *DOLPP1* cause fatal Type I congenital disorders of glycosylation in humans (69), and *DOLPP1* cDNA can complement growth and glycosylation defects in a *S. cerevisiae cwh8Δ* mutant (68), highlighting its conserved functional role in glycosylation across eukaryotes. Given this strong conservation, the presence of *CWH8* alone does not fully explain the distinct capacity of *C. albicans* to produce farnesol; rather, differences in its regulation or enzymatic activity likely influence farnesol production. Our data reveal that expression of the *CWH8* homolog from *C. lusitaniae*, a species that does not naturally secrete farnesol, fails to restore farnesol production in a *C. albicans cwh8ΔΔ* mutant, although it rescues cell wall integrity (Fig. 6F and G). This suggests that species-specific factors or differences in Cwh8 enzymatic activity or substrate specificity critically influence farnesol biosynthesis in *C. albicans*.

It is well established that *C. albicans* and closely related species normally secrete farnesol (70), whereas humans and most fungi do not secrete it under standard conditions. Moreover, farnesol production in many fungi requires inhibition of the ergosterol biosynthesis pathway (71), while it is readily detected in *C. albicans* supernatants without inhibition (12, 13, 70). This difference could be explained by at least two possibilities: farnesol is metabolized differently by mammalian cells and *C. albicans* (27, 72). Free farnesol can be used for cholesterol biosynthesis and protein prenylation by mammalian cells (73), while *C. albicans* lacks the ability to salvage farnesol for ergosterol metabolism (72). Thus, *C. albicans* may have evolved mechanisms to secrete endogenously accumulated farnesol due to the cytotoxic effects of either FPP (63, 64) or farnesol, possibly avoiding the toxic effects of excess levels of their precursor IPP (65). On a related note, *C. albicans* is more tolerant of exogenous farnesol than other eukaryotes (23, 24, 74), even other *Candida spp.* (75). This tolerance suggests active mechanisms that provide farnesol tolerance. Regulation of flux through the sterol biosynthetic pathway and the FPP/FP to farnesol pathway is different in *C. albicans*. A key metabolic feature of farnesol-producing yeasts is the dramatically elevated farnesyl phosphate (FP) pool relative to farnesyl pyrophosphate (FPP) (Fig. 6A through C). Yeast species that do not secrete farnesol under physiologic conditions (*C. lusitaniae* and *S. cerevisiae*) lack this elevated FP/FPP ratio (Fig. 6C). This significant FP accumulation appears essential for robust farnesol secretion and can be experimentally upregulated by ERG gene mutations or chemical inhibition of sterol biosynthesis (71). Notably, the FP pool in *C. albicans* can reach such high levels partly because of the lack of a farnesol salvage pathway (72). This increase in FP pool size in farnesol-producing species is likely driven by Cwh8 activity, which could be influenced by multiple factors, including species-specific variations in the enzymatic activity or substrate specificity of Cwh8; regulatory factors that modulate Cwh8 expression or function; or accessory proteins that affect substrate availability and enzyme efficiency. Together, these regulatory and metabolic differences, in concert with species-specific variations in Cwh8 enzymatic activity, enable *C. albicans* to maintain high intracellular levels of farnesol precursors and secrete farnesol constitutively, distinguishing its physiology from non-farnesol-producing yeasts.

### Cwh8 as a tool to probe farnesol’s role in cell physiology and pathogenesis

 *cwh8ΔΔ* is the first farnesol negative mutant of *C. albicans,* and thus it should prove useful in exploring farnesol’s role in pathogenesis. However, the dual nature of the mutant, defects in farnesol synthesis and cell wall integrity, means we cannot distinguish which defect is responsible for an observed phenotype. Fortunately, this obstacle has been overcome by complementing *cwh8ΔΔ* with *CWH8* from *C. lusitaniae*, a species lacking farnesol secretion. This construct remedies any cell wall defects, but it doesn’t restore farnesol production. Given the direct relationship between *CWH8* expression and farnesol production for *C. albicans*, mutants with regulatable *CWH8* expression (i.e., regulatable promoters) and the *cwh8ΔΔ::CLUG_01716* strain should be powerful tools for dissecting the multifaceted roles of endogenous farnesol. Such tools would allow investigations into farnesol’s contributions to commensal fitness in host environments such as the microbiome of the mouth and intestine; disseminated infection; *Candida* interspecies interactions; fundamental fungal physiology, including the role of farnesol in protein prenylation and yeast-mycelial dimorphism (27).

## MATERIALS AND METHODS

### Strains and media

All strains used in this study are listed in Table S1. Strains were maintained in 15% glycerol stocks stored at −80°C. Strains were routinely grown in yeast extract-peptone-dextrose (YPD) (2% Bacto Peptone, 2% dextrose, and 1% yeast extract) plates and grown at 30°C. For farnesol assays, strains were grown in YPD, mRPMI (RPMI 1640 with glutamine, without bicarbonate, 2% glucose, buffered with 0.165 mol/L MOPS [3-(N-morpholino) propanesulfonic acid] (M9381, Sigma) supplemented with GPP mineral stock [13, 42]), Lee’s medium (43) or the defatted albumin medium (44). For germ tube assays, resting cells were prepared in modified glucose-salts-biotin media (mGSB) (12) and inoculated into glucose-proline-phosphate media (GPP) (42). For embedded filamentation assays, yeast extract-peptone-sucrose agar was used (76).

The transcription regulator (TR) mutants were obtained from the Fungal Genetic Stock Center. The TR mutants were constructed from *C. albicans* SN152, an auxotroph for Arg, His, and Leu (37), and each of the TR mutants is auxotrophic for Arg (37). The strain SN152^+^ was created by reintroducing a single allele of *HIS1* and *LEU2* into the SN152 parent (37) and is used as a control for the TR strains in this study.

### Assessment of farnesol accumulation

 Farnesol measurements were performed as previously described (13, 38). Briefly, strains were inoculated from YPD plates into 3 mL of media containing either YPD or mRPMI and grown overnight at 30°C. This preinoculum was diluted 1:100 (~0.2 OD_600_) into 75 mL of either YPD or mRPMI of culture in 250 mL flasks grown at 30°C for up to 72 hours, 225 RPM. Ten milliliter cultures were extracted with 2 mL of ethyl acetate containing an internal standard (1-tetradecanol). Extracts were analyzed on an Agilent Technologies 7890B gas chromatography system (Santa Clara, CA, USA) equipped with autosampler and flame ionization detector (FID). Chromatographic separation was performed on an HP-Innowax (Agilent 19091 N-133I) column. Two microliters of each sample was injected (splitless) with hydrogen as carrier gas at an initial temperature of 90°C ramped at a rate of 30°C/min to a final temperature of 245°C and held for 7 min. Data files were batched and analyzed using OpenLab Services (Agilent Technologies) with software-calculated response factors based on standard calibrator solutions (13).

### Antifungal and stress assays

 The susceptibility to various stressors was determined by serial dilution growth tests, as described by Petropavlovskiy et al. with modifications (77). YPD plates were prepared separately with 20 µM Calcofluor white, and 280 µM Congo red. SD plates were prepared separately with 100 µM fluconazole or 8.2 µM terbinafine. Overnight cultures were grown in 3 mL YPD and diluted to an OD_600_ of 1. From this, tenfold dilutions were generated, and 5 µL of each dilution was spotted onto the relevant YPD or SD +Arg + Leu plates with and without stressor. Growth was captured with a UVP Chemstudio imager (Analytik Jena) after 48 hours of incubation at 30°C.

 Two methods were used in parallel to determine antifungal susceptibility. For the Etest method (BioMérieux), overnight YPD grown cultures were adjusted to an optical density (OD_600_) of 0.5 in sterile water and 100 µL spread onto either YPD or RPMI plates. The test strip was placed onto the lawn of cells using sterile forceps and incubated at 30°C for 48 h. Broth microdilution assays were performed according to the Clinical Laboratory and Standards Institute (CLSI) standard method (M27-A3) (78). Plates were incubated at 30°C for 48 h. To determine fungicidal activity, 5 µL of the 48 h broth microdilution assays were spotted onto YPD agar and incubated for 24 h.

### Statistical analysis

 Statistical analyses were performed using Microsoft Excel (Version 16.97, Microsoft Office, Las Vegas, NV, USA) and GraphPad Prism Software (Version 10.4.1, San Diego, CA, USA). Data are represented as mean ± SD of at least three biological replicates unless otherwise stated. Differences between groups that were normally distributed and homoscedastic were assessed by one-way ANOVA with Dunnett’s multiple comparisons test. Differences between groups that were not normally distributed or heteroscedastic were assessed by the Kruskal–Wallis test with Dunn’s multiple comparisons tests. Both analyses were performed in GraphPad Prism. Differences were considered significant at *P* < 0.05 (*P < 0.05, **P < 0.01, and ***P < 0.001).

### Additional methods

See Text S1 in the supplemental material for methods describing strain construction, quantitation of farnesyl monophosphate and pyrophosphate, embedded growth and filamentation assays, RNA-sequencing of *msn4ΔΔ*, *cas5ΔΔ, cwh8ΔΔ, cwh8ΔΔ::pTDH3-CWH8,* and 50 µM farnesol-treated cells, and measurement of *CWH8* gene expression.

## Data Availability

Strains are listed in Table S1 and are available upon request. The RNA-seq raw data have been deposited in NCBI’s Gene Expression Omnibus (79) and are accessible through GEO Series accession number GSE289051. Table S1 contains strains and oligonucleotides used in this study. Table S2 contains *cas5ΔΔ* and *msn4∆∆* differentially expressed genes. Table S3 contains results from the *cas5∆∆*, *msn4∆∆*, *swi4∆∆*, and *ahr1∆∆* gene overlap analysis. Table S4 contains *cwh8ΔΔ*, *cwh8ΔΔ::pTDH3-CWH8*, and 50 µM FOH differentially expressed genes. Table S5 contains results from the *cwh8ΔΔ*, *cwh8ΔΔ::pTDH3-CWH8*, and 50 µM FOH gene overlap analysis. Table S6 contains results from the *cwh8ΔΔ*, *cwh8ΔΔ::pTDH3-CWH8*, and 50 µM FOH GO analysis.

## SUPPLEMENTAL MATERIAL

Text S1 mbio.02290-25-s0001.pdf Additional methods.

Supplemental figures mbio.02290-25-s0002.pdf Fig. S1 to S6.

Table S1 mbio.02290-25-s0003.xlsx Strains, plasmids, and oligonucleotides.

Table S2 mbio.02290-25-s0004.xlsx *cas5* and *msn4* differentially expressed genes.

Table S3 mbio.02290-25-s0005.xlsx *cas5*, *msn4*, *swi4*, and *ahr1* gene overlap analysis results.

Table S4 mbio.02290-25-s0006.xlsx *cwh8*ΔΔ, *cwh8*ΔΔ::*pTDH3-CWH8*, and 50 μM FOH differentially expressed genes.

Table S5 mbio.02290-25-s0007.xlsx *cwh8*ΔΔ, *cwh8*ΔΔ::*pTDH3-CWH8*, and 50 μM FOH gene overlap results.

Table S6 mbio.02290-25-s0008.xlsx *cwh8*ΔΔ, *cwh8*ΔΔ::*pTDH3-CWH8*, and 50 μM FOH enriched GO pathways

ASM does not own the copyrights to Supplemental Material that may be linked to, or accessed through, an article. The authors have granted ASM a non-exclusive, world-wide license to publish the Supplemental Material files. Please contact the corresponding author directly for reuse.
