# Supplementary material for: Cwh8 moonlights as a farnesyl pyrophosphate phosphatase and is essential for farnesol biosynthesis in Candida albicans
Source: mBio. 2025 Sep 8;16(10):e02290-25. doi: 10.1128/mbio.02290-25 (PMC12505894; doi:10.1128/mbio.02290-25)
Supplement: Text S1 — Additional methods. [file mbio.02290-25-s0001.pdf]

Generation of *C. albicans* pyrophosphatase null mutants using CRISPR-Cas9

The LEUpOUT CRISPR-Cas9 system (1) was used to generate deletion strains of the *DPP1, DPP2*, *DPP3*, and *CWH8* genes in *C. albicans*. Oligonucleotides and plasmids are listed in Supplementary Table S1. Guide RNAs (gRNA) were designed in Benchling (Biology Software) to target the open reading frame (ORF) with an on/off-target score greater than 60 with the following parameters: (Design Type, single guide; Guide Length, 20bp; Genome, CA22 (*Candida albicans* SC5314 (diploid)); PAM, NGG). This guide was used to generate a “C” fragment specific for each gene of interest by Cloning-free stitching (1). The repair template for deletion of each ORF was constructed from two oligonucleotide primers designed to have homology to the 5’ UTR and 3’ UTR of each gene and included a Add Tag sequence (GG) to introduce a PAM recognition sequence for reconstitution. Transformation of the *C. albicans* was performed by lithium acetate method (1) and positive transformants were selected on YPD with 200 µg/ml nourseothricin. The CRISPR-Cas9 cassette was recycled from confirmed mutants by plating onto either synthetic defined medium (SD) or SD plus 40 µg/mL arginine depending on the auxotrophic requirements of the parent strain. Mutants were screened by PCR with ORF and flank specific primers. Successful deletion of each gene was confirmed by loss of a product for the ORF specific primer and a truncated product for the flank specific primer (corresponding to to the ORF deletion).

Generation of *CWH8* reconstitution and p*TDH3-CWH8* mutants using CRISPR-Cas9

To generate a wild-type *CWH8* reconstituted strain, a repair template with wild-type *CWH8* with 569 bp upstream and 541 bp downstream homology was amplified from SC5314 genomic DNA using primers oAA1021 and oAA1022 (Table S1) by PCR with Phusion Plus

Master Mix (Thermofischer). This product was used as the repair template along with a guide gDNA targeting at the introduced Add Tag PAM sequence. Successful reconstitution was confirmed by the presence of a product for the ORF specific primer and a wild-type product for the flank specific primer.

To generate the *CWH8* constitutive mutant an overlap extension PCR strategy was used. First, the *TDH3* promoter DNA flanked by *CWH8* sequences (position -868 to-1) was amplified from SC5314 DNA using long oligonucleotides containing homology to the *CWH8* promoter (position -519) on the forward primer (oAA1064) and homology to the *CWH8* CDS (position +1 - +29) on the reverse primer (oAA1065). Second, the full length *CWH8* coding region plus downstream homology was amplified from SC5314 genomic DNA using long oligonucleotides containing homology to p*TDH3* on the forward primer (oAA1066) and the reverse primer (oAA1022) used for reconstitution above. 1µL of each product from above was pooled and combined using an overlap extension PCR reaction. The product was verified on a gel for correct size, pooled, and used as the repair template along with a guide gDNA targeting the introduced Add Tag PAM sequence above.

Functional Complementation of *Candida albicans cwh8ΔΔ* with *C. lusitaniae CWH8*

To generate a *C. albicans* strain with the *C. lusitaniae CWH8* transgene, a repair template with the wild-type *C. lusitaniae CWH8* homolog (CLUG_01716) was amplified from *C. lusitaniae* U04 genomic DNA using primers oDG0017 and oDG0018 (Table S1) by PCR with Phusion Plus Master Mix (Thermofischer). This product was used in a subsequent PCR reaction using oDG0019 and oDG0020 to add 50bp of *C. albicans CWH8* upstream and downstream homology. This product was used as the repair template for *C. albicans cwh8ΔΔ* along with a

guide gDNA targeting at the introduced Add Tag PAM sequence. Successful reconstitution was confirmed by the presence of a product for the ORF specific primer and a wild-type product for the flank specific primer.

Filamentation assays

Germ tube assays. Resting cells were prepared by growing each strain in modified glucose salts biotin media with supplemented arginine and leucine (mGSB+Arg+Leu) for 48 hours. These cultures were washed four times with Dulbecco's phosphate-buffered saline (DPBS, pH 7.0) and resuspended in 10mL DPBS for storage at 4°C. A density of 5 x 10^5 cells/mL of resting cells were seeded in 1mL prewarmed GPP media containing arginine and leucine or RPMI 1640. A 100 µL sample was added to a clear flat-bottomed 96-well plate and incubated at 37 °C for GPP or a 5% CO_2 incubator at 37 °C for RPMI 1640. The morphology of each strain was assessed at 4 hours post-inoculation using a EVOS FL inverted microscope (Invitrogen). One representative field was captured for each well and assessed for the percentage of filamentous cells. The morphology of each cell in the captured fields was defined as either yeast or filamentous cells. Images are representative of three independent experiments.

Biofilm Formation. The biofilm formation assay was performed as previously described (2). Briefly, overnight cultures were prepared in YPD at 30°C. This overnight culture was inoculated at a density of 5 x 10^5 cells/mL into 1 mL prewarmed RPMI 1640 medium (Gibco, Cat: 11875101). 200 µL of this suspension was added to flat bottomed 96-well plate in five technical replicates and incubated at 37°C, 5% CO_2. After 90 minutes wells were washed with PBS and fresh media was added back to the wells and incubated for an additional 24 hours. The resulting biofilms were examined at 6 hr, 12hr, 18hr, and 24 hr post inoculation using a EVOS

FL inverted microscope (Invitrogen). Images are representative across three independent experiments.

Embedded Filamentation. The embedded filamentation assay was performed as previously described (3). Briefly, YPD pregrown cells were submersed in molten YPS agar and grown for 5 days at 25°C. Embedded filamentation score was determined from >150 colonies/strain across 3 independent experiments and colonies were scored from 0 (no filamentation) to 4 (extensive filamentation) using criteria presented by Azadmanesh et al. (4). Hyphal length measurements were performed using Fiji (5) to measure >100 hyphae/strain. If a cell had no visible hyphae, zero hyphal length was recorded instead. Images are representative across three independent experiments.

Quantitation of Farnesyl Monophosphate and Pyrophosphate

Materials and standards. Acetonitrile and methanol were LC-MS grade obtained from EMD Biosciences (San Diego, CA, USA). Hexanes were optima grade and isopropanol was HPLC grade both obtained from Fisher Scientific (Pittsburgh, PA, USA). Ammonium hydroxide solution ≥ 99.99% trace metal basis, ammonium carbonate 99.999% trace metal basis, and methylamine solution (33% in absolute ethanol) were purchased from Sigma Aldrich (St. Louis, MO, USA). Standards for quantification of farnesyl monophosphate (FP), surrogate R-citronellyl pyrophosphate (CPP), and internal standard R-citronellyl monophosphate (CP) were purchased from Isoprenoids LLC (Tampa, FL, USA). Standard used for farnesyl pyrophosphate quantification was purchased from Sigma (St. Louis, MO, USA).

Mass spectrometry. Standards were dissolved at 2 mM stock in methanol and stored at -20 °C. Stock standards were diluted to 20 µM in 1 mL mobile phase A (20% methanol in water, 10 mM ammonium carbonate, 0.125% ammonium hydroxide) and directly infused into the TurboIonSpray source of a Sciex 4000 QTRAP LC-MS/MS System (Applied Biosystems, Foster City, CA, USA) using KDS100 syringe pump (KD Scientific inc., Holliston, MA, USA) at 10 µL/ min. The source temperature was set to 100 °C, IonSpray Voltage set at -4200V, curtain gas at 10 psi, nebulizing gas (GS1) at 20 psi, focusing gas (GS2) at 0 psi, and interface heater engaged. Declustering potential (DP) and collision energy (CE) were optimized on a compound dependent basis (TextS1 Table 1).

TextS1 Table 1: LC-MSMS analyte characteristics

Analyte	MRM Transition	DP	CE	RT
Citronellyl-P (CP)	235.2 → 79.1	-70	-30	5.92
Citronellyl-PP (CPP)	314.9 → 79.1	-70	-30	5.55
Farnesyl-P (FP)	301.0 → 79.1	-70	-30	7.58
Farnesyl-PP (FPP)	381.1 → 79.1	-70	-30	7.06

Sample preparation and extraction. Triplicate mRPMI cultures (50 mL) were inoculated at 0.2 OD_{600} from isolated colonies on YPD plates and grown overnight in 250 mL flasks. Whole culture sample volume of 100 OD_{600} equivalents (e.g. 10 mL of a 10 OD_{600} culture) were collected in 35 mL glass centrifuge tubes in duplicate and lyophilized using FTS Systems Flexi-Dry MP freeze dryer (Stone Ridge, NY, USA) for approximately 20 hours. A third whole culture aliquot equivalent to the volume harvested above was centrifuged, decanted and pellet was lysed in 1 mL RIPA buffer with bead beating for protein quantitation by BCA assay for metabolite normalization. The lyophilized samples were reconstituted in 3 mL of extraction solvent (lower phase of isopropanol/ hexane/ water (55:20:25) v/v/v) with approximately 300 µL of 425-600 µm acid washed glass beads (Sigma). Stock CPP was diluted

1:10 in extraction solvent and 10 µL was added as surrogate to each sample. Samples were fully homogenized with vigorous vortexing in 30 second intervals with intermittent incubation/ sonication in 50 °C water bath six rounds for a total of 3 minutes of bead beating. Fully homogenized samples were centrifuged at 500 g for 10 minutes. After centrifugation the supernatant was transferred to a second tube and the pellet was extracted once more with 3 mL extraction solvent. The supernatants were pooled and dried under a stream of nitrogen. The dried, crude extract was dissolved in 2 mL of 33% methylamine solution in ethanol/ water (7:3 v/v) and incubated at 50 °C for 1 hour for deesterification. After hydrolysis the samples were again dried under a stream of nitrogen and dissolved in 500 µL methanol with 4 µM internal standard CP (resuspension solution) by vortexing, heating, and sonication. Samples were again centrifuged at 500 g for 10 minutes to remove any insoluble debris and transferred to autosampler vial containing inserts. The samples were also diluted 1:100 in 1 mL of resuspension solution using 10 µL Hamilton syringe. Both undiluted and 1:100 dilution samples were analyzed by LC-MS/MS.

Liquid Chromatographic and MS/MS Conditions. Sample analysis was performed using a Shimadzu Prominence HPLC system (ABSciex, Framingham, MA, USA) equipped with two pumps (LC-20AD XR), a column oven (CTO-20A), and autosampler (SIL-20A/CXR) fitted with a 50 µL sample loop, 100 µL X-type syringe, with water/methanol/acetonitrile/isopropanol (1:1:1:1 v/v/v/v) wash solution. Chromatographic conditions and MRM transitions for mevalonate pathway pyrophosphates previously reported were reproduced with modifications and additions (6). Flow from the sample injector led to an ACCQ-TAG Ultra C18 column (100 mm x 2.1 mm I.D., 1.7 µm particle size, Water, Milliford, MA, USA) equipped with a C18 guard column held at 40 °C. Ten µL of sample was injected and eluted at a flow rate of 0.20 ml min^{-1}

with a binary gradient system consisting of mobile phase A, 20% methanol in water, 10 mM ammonium carbonate + 0.125% ammonium hydroxide, and mobile phase B, acetonitrile: methanol (3:1 v/v) + 0.125% ammonium hydroxide (see Text Table S2 for gradient details). The initial eluate was directed via a valco valve to waste for 1 minute after which the flow was directed to the TurboIonSpray source of the 4000 QTRAP mass spectrometer (ABSciex, Framingham, MA, USA). The probe was vertically positioned at 2 mm and charged with -4200 V. The temperature was held at 450 °C, GS1 was set at 40 psi, GS2 at 50 psi, curtain gas at 10 psi, and the interface heater was engaged.

TextS1 Table 2: Mobile phase gradient

Time	% B
0	0
1.0	0
7.0	65
9.0	65
9.5	95
11.5	95
11.6	0
13.6	0

Standard curve generation, data collection, and analysis. Data was collected with Analyst 1.7.1 software (Applied Biosystems). Serial 2-fold dilutions were constructed in triplicate for FP and FPP starting from 11.5 pmol/ µL in sample resuspension solution (4 µM CP in methanol). Centroided peak area was used to calculate the analyte to internal standard ratio of each dilution and plotted as pmol of analyte per 500 µL sample resuspension volume. Experimental ratios were plotted against these graphs to establish sample analyte quantity in pmol. This quantity of analyte in pmol was then divided by mg of protein in the sample to yield a value of pmol of metabolite per mg of protein.

RNA-sequencing of ***msn4*** and ***cas5***

RNA preparation. Three biological replicates of SN152+, *msn4*, and *cas5* were grown in YPD, 16 hours and 30°C, 225 RPM to generate a preinoculum. This preinoculum was diluted into 75 mL of YPD (0.1 OD_{600}). Cultures were harvested after they had reached an early stationary phase (10 OD_{600}), which is when farnesol levels peak (7), washed in ice-cold RNase free $H_2 0$, and frozen at -80°C. Total RNA was extracted using the hot-phenol method previously described by our group (8) and further cleaned up using the RNeasy mini kit (Qiagen). RNA quality was assessed by gel, spectrophotometric analysis (Spectramax Plus 384, Molecular Devices), and bioanalyzer analysis (Agilent). RNA samples sent for sequencing had a RIN > 8 and absorbance 260/280 of >2.0. RNA was quantified for sequencing by Quibit (Thermofischer Scientific).

RNA sequencing and data analysis. For RNA-seq, three biological replicates of SN152+, *msn4*, and *cas5* total RNA was sent to Seqcenter (Pittsburgh, PA) to perform RNA sequencing and differential expression analysis. Briefly, samples were treated with DNase (RNAse free); Invitrogen. Library preparation was performed using Illumina's Stranded mRNA prep with 10bp IDT for Illumina indices. Sequencing was performed on a NovaSeq6000 giving 2x51bp reads. Demultiplexing, quality control, and adapter trimming was performed with bcl-convert (Version 4.0.3) (9). Sequencing statistics are included in Table S3 with all samples having a 94% of base pairs above >Q30. Read mapping was performed via STAR (Version 2.7.10a) (10). Feature quantification was performed using RSEM (Version 1.3.3) (11). Read counts loaded into R (12) (Version 4.0.2) and were normalized using edgeR's (Version 1.14.5) (13) Trimmed Mean of M values (TMM) algorithm. Subsequent values were then converted to counts per million (cpm). Differential expression analysis was performed using edgeR's Quasi-

Linear F-Test (qlfTest) functionality against treatment groups. Results of the qlfTest for all genes in addition to the normalized counts per million are found in Table S3. Differentially expressed genes with $|log2FC| > 1$ and $p < .05$ can be found in Table S2. Volcano plots were generated in Graphpad Prism (Version 10.2.3) after analysis and annotation in R (Version 4.3.2) (12) using the tidyverse package (14) Finally, pathway analysis was performed using limma's (Version 3.44.3) (15) "kegga" functionality.

RNA-sequencing of *cwh8ΔΔ*

RNA preparation. Four biological replicates of MAY1375 and *cwh8ΔΔ* were grown in mRPMI 1640, 16 hours and 30°C, 225 RPM to generate a preinoculum. This preinoculum was diluted into 50 mL of mRPMI 1640 (0.1 OD_{600}) and grown 30°C. Cultures were harvested after 6 hrs, washed in ice-cold RNase free H_20, and frozen at -80°C. Total RNA was extracted using TRIzol (Thermofischer) and further cleaned up using the RNeasy mini kit (Qiagen). RNA quality was assessed by gel, spectrophotometric analysis (Spectramax Plus 384, Molecular Devices), and bioanalyzer analysis (Agilent). RNA samples sent for sequencing had a RIN > 8 and absorbance 260/280 of >2.0. RNA was quantified for sequencing by Quibit (Thermofischer Scientific).

RNA sequencing and data analysis. For RNA-seq, four biological replicates of MAY1375 and *cwh8ΔΔ* total RNA were sent to SeqCoast (Portsmouth, NH) to perform RNA sequencing and differential expression analysis. Briefly, samples were treated with DNase (RNAse free); Invitrogen. Library preparation was performed using Illumina's Stranded mRNA prep with Illumina dual indexes. Sequencing was performed on a NovaSeq 2000 giving 2x150bp reads. Quality control, and adapter trimming was performed with Trimmomatic (version 0.39)

Read mapping and feature quantification was performed using Kallisto (Version 2.1.2) (16). Read counts loaded into R (12) (Version 4.0.2) and differential expression analysis was performed using DESeq2 (Version 2.23.0) (17). Differentially expressed genes with |log2FC| > 1 and p < .05 can be found in Table S4. Volcano plots were generated in in R (Version 4.3.2) (12) using the EnhancedVolcano package (Version 2.3) (18).

RNA-sequencing of *cwh8ΔΔ::pTDH3-CWH8* and 50 µM farnesol treated cells.

RNA preparation. Eight biological replicates of MAY1375 and *cwh8ΔΔ::pTDH3-CWH8* were grown in mRPMI 1640, 16 hours and 30°C, 225 RPM to generate a preinoculum. This preinoculum was diluted into 30 mL of mRPMI 1640 (0.1 OD_{600}) with and without 50 µM *E,E*-farnesol and grown 30°C. Cultures were harvested after 6 hrs, washed in ice-cold RNase free $H_2$0, and frozen at -80°C. Total RNA was extracted using TRIzol (Thermofischer) and further cleaned up using the RNeasy mini kit (Qiagen). RNA quality was assessed by gel, spectrophotometric analysis (Spectramax Plus 384, Molecular Devices), and bioanalyzer analysis (Agilent). RNA samples sent for sequencing had a RIN > 8 and absorbance 260/280 of >2.0. RNA was quantified for sequencing by Quibit (Thermofischer Scientific).

RNA sequencing and data analysis. For RNA-seq, four biological replicates of MAY1375, MAY1375 + 50µM E,E-farnesol, and *cwh8ΔΔ::pTDH3-CWH8* total RNA were sent to the Genomics and Molecular Biology Shared Resource at Dartmouth (Dartmouth, NH) to perform RNA sequencing. RNA was hybridized to FastSelect probes (Qiagen) for ribodepletion, followed by library preparation using the Kapa RNA HyperPrep kit (Roche) following manufacturer's instructions. Sequencing was performed on a NovaSeq2000 giving 2x50bp reads.

Read mapping and feature quantification was performed using Kallisto (Version 2.1.2) (16). For downstream analysis, scripts were adapted from Berry et al. (19). Read counts loaded into R (12) (Version 4.0.2) and differential expression analysis was performed using DESeq2 (Version 2.23.0) (17). Differentially expressed genes can be found in Table S4. Volcano plots were generated in in R (Version 4.3.2) (12) using the EnhancedVolcano package (Version 2.3) (18).

Measurement of *CWH8* gene expression

RNA preparation. Three biological replicates of SN152+, *swi4*, *msn4*, *rap1* and *ahr1* were grown in YPD, 16 hours and 30°C, 225 RPM to generate a preinoculum. This preinoculum was diluted into 25 mL of YPD (0.2 OD_{600}). Cultures were harvested after they had reached an early stationary phase (10 OD_{600}), washed in ice-cold RNase free H_20, and frozen at -80°C. Total RNA was extracted using the hot-phenol method (8) and further cleaned up using the RNeasy mini kit (Qiagen, Cat. No. 74104). RNA quality was assessed by spectrophotometric analysis (Spectramax Plus 384, Molecular Devices), and integrity was confirmed by agarose gel electrophoresis.

cDNA preparation. 1 µg of total RNA was reverse transcribed into cDNA using the iScript™ gDNA Clear cDNA Synthesis Kit (Bio Rad, Cat. No. 1725034), according to the manufacturer's instructions. Reactions were first incubated at 25°C for 5 minutes and 75°C for 5 minutes with DNase to remove genomic DNA. Reactions were then incubated at at 25°C for 5 minutes, 46°C for 20 minutes and 95°C for 1 minute to synthesize cDNA.

RT-qPCR. RT-qPCR was performed using SsoAdvanced™ Universal SYBR® Green Supermix (Bio Rad, Cat. No. 1725271) on a CFX96 Deep Well PCR System (Bio Rad). CWH8 primers were designed using Primer3 software and synthesized by Integrated DNA

Technologies. Each reaction was performed in a total volume of 20 µL, containing 10 µL SYBR Green mix, 0.5 µM of each primer, and 20 ng of cDNA. The qPCR cycling conditions were as follows: 95°C for 2 minutes, followed by 40 cycles of 95°C for 15 seconds and 60°C for 1 minute. A melt curve analysis was performed to confirm specificity

Data analysis. Relative expression levels were normalized to the housekeeping gene ACT1. Relative gene expression was calculated using the $2^{(-\Delta\Delta Ct)}$ method (20). All qPCR reactions were performed in technical triplicate, with at least three biological replicates per condition. Primer specificity was confirmed by melt curve analysis.

References

1. Nguyen N, Quail MMF, Hernday AD. 2017. An Efficient, Rapid, and Recyclable System for CRISPR-Mediated Genome Editing in Candida albicans. mSphere 2:e00149-17.

2. Gulati M, Lohse MB, Ennis CL, Gonzalez RE, Perry AM, Bapat P, Arevalo AV, Rodriguez DL, Nobile CJ. 2018. In Vitro Culturing and Screening of *Candida albicans* Biofilms. Curr Protoc Microbiol 50:e60.

3. Naseem S, Douglas LM, Konopka JB. 2020. Candida albicans Agar Invasion Assays. Bio Protoc 10:e3730.

4. Azadmanesh J, Gowen AM, Creger PE, Schafer ND, Blankenship JR. 2017. Filamentation Involves Two Overlapping, but Distinct, Programs of Filamentation in the Pathogenic Fungus Candida albicans. G3 (Bethesda) 7:3797–3808.

5. Schindelin J, Arganda-Carreras I, Frise E, Kaynig V, Longair M, Pietzsch T, Preibisch S, Rueden C, Saalfeld S, Schmid B, Tinevez J-Y, White DJ, Hartenstein V, Eliceiri K,

Tomancak P, Cardona A. 2012. Fiji: an open-source platform for biological-image analysis. Nat Methods 9:676–682.

6. Chhonker YS, Haney SL, Bala V, Holstein SA, Murry DJ. 2018. Simultaneous Quantitation of Isoprenoid Pyrophosphates in Plasma and Cancer Cells Using LC-MS/MS. Molecules 23:3275.

7. Boone CHT, Gutzmann DJ, Kramer JJ, Atkin AL, Nickerson KW. 2022. Quantitative assay for farnesol and the aromatic fusel alcohols from the fungus *Candida albicans*. Appl Microbiol Biotechnol https://doi.org/10.1007/s00253-022-12165-w.

8. Kebaara BW, Baker KE, Patefield KD, Atkin AL. 2012. Analysis of Nonsense-Mediated mRNA Decay in *Saccharomyces cerevisiae*. Current Protocols in Cell Biology 54.

9. bcl-convert: A proprietary Illumina software for the conversion of bcl files to basecalls. (4.0.3).

10. Dobin A, Davis CA, Schlesinger F, Drenkow J, Zaleski C, Jha S, Batut P, Chaisson M, Gingeras TR. 2013. STAR: ultrafast universal RNA-seq aligner. Bioinformatics 29:15–21.

11. Li B, Dewey CN. 2011. RSEM: accurate transcript quantification from RNA-Seq data with or without a reference genome. BMC Bioinformatics 12:323.

12. R Core Team. 2021. R: A language and environment for statistical ## computing. R Foundation for Statistical Computing.

13. Robinson MD, McCarthy DJ, Smyth GK. 2010. edgeR : a Bioconductor package for differential expression analysis of digital gene expression data. Bioinformatics 26:139–140.

14. Wickham H, Averick M, Bryan J, Chang W, McGowan L, François R, Grolemund G, Hayes A, Henry L, Hester J, Kuhn M, Pedersen T, Miller E, Bache S, Müller K, Ooms J, Robinson D, Seidel D, Spinu V, Takahashi K, Vaughan D, Wilke C, Woo K, Yutani H. 2019. Welcome to the Tidyverse. JOSS 4:1686.

15. Ritchie ME, Phipson B, Wu D, Hu Y, Law CW, Shi W, Smyth GK. 2015. limma powers differential expression analyses for RNA-sequencing and microarray studies. Nucleic Acids Research 43:e47–e47.

16. Bray NL, Pimentel H, Melsted P, Pachter L. 2016. Near-optimal probabilistic RNA-seq quantification. Nat Biotechnol 34:525–527.

17. Love MI, Huber W, Anders S. 2014. Moderated estimation of fold change and dispersion for RNA-seq data with DESeq2. Genome Biol 15:550.

18. Blighe K, Rana S, Lewis K. EnhancedVolcano: Publication-ready volcano plots with enhanced colouring and labeling.

19. Berry ASF, Farias Amorim C, Berry CL, Syrett CM, English ED, Beiting DP. 2021. An Open-Source Toolkit To Expand Bioinformatics Training in Infectious Diseases. mBio 12:e0121421.

20. Livak KJ, Schmittgen TD. 2001. Analysis of Relative Gene Expression Data Using Real-Time Quantitative PCR and the $2^{-\Delta\Delta CT}$ Method. Methods 25:402–408.
