# Supplementary material for: Cwh8 moonlights as a farnesyl pyrophosphate phosphatase and is essential for farnesol biosynthesis in Candida albicans
Source: mBio. 2025 Sep 8;16(10):e02290-25. doi: 10.1128/mbio.02290-25 (PMC12505894; doi:10.1128/mbio.02290-25)
Supplement: Supplemental figures — Fig. S1 to S6. [file mbio.02290-25-s0002.pdf]

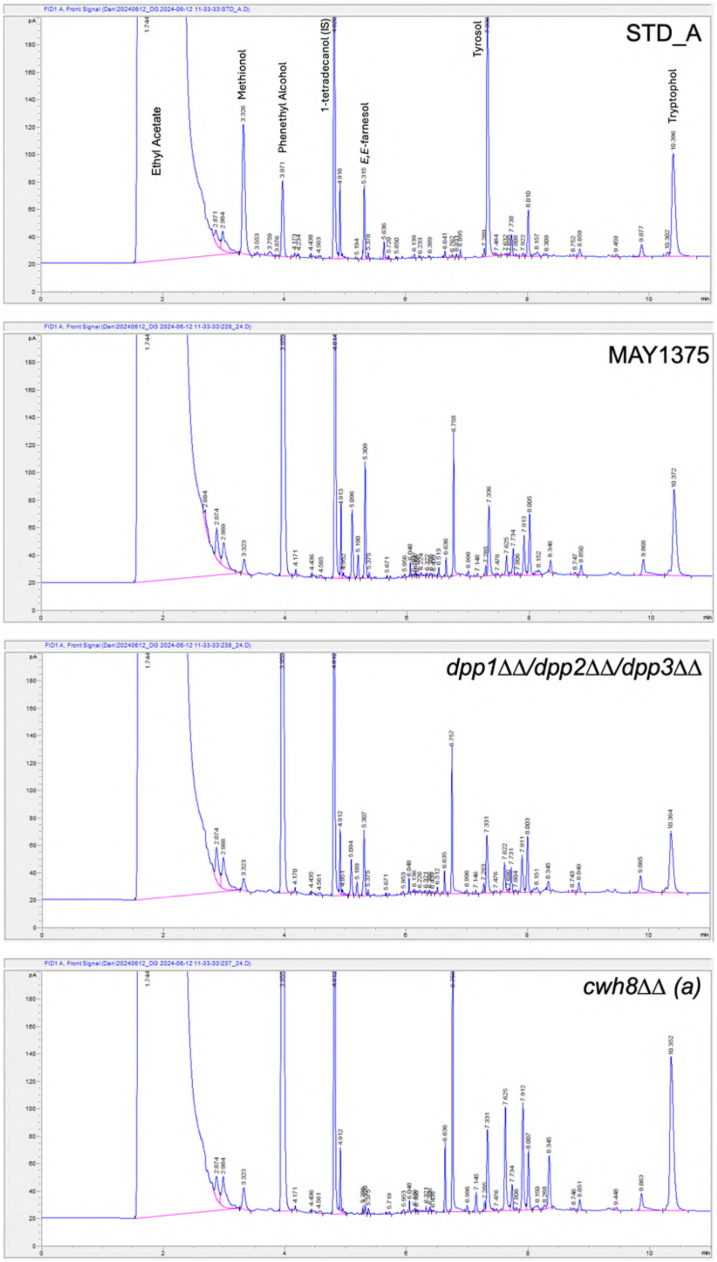

Figure S1. Representative gas chromatographs of ethyl acetate extracts of a known standard (Standard A, Boone et al., 2022), MAY1375, dpp1/dpp2/dpp3*ΔΔ*, and the *cwh8ΔΔ* grown in YPD at 24 hours post-inoculation.

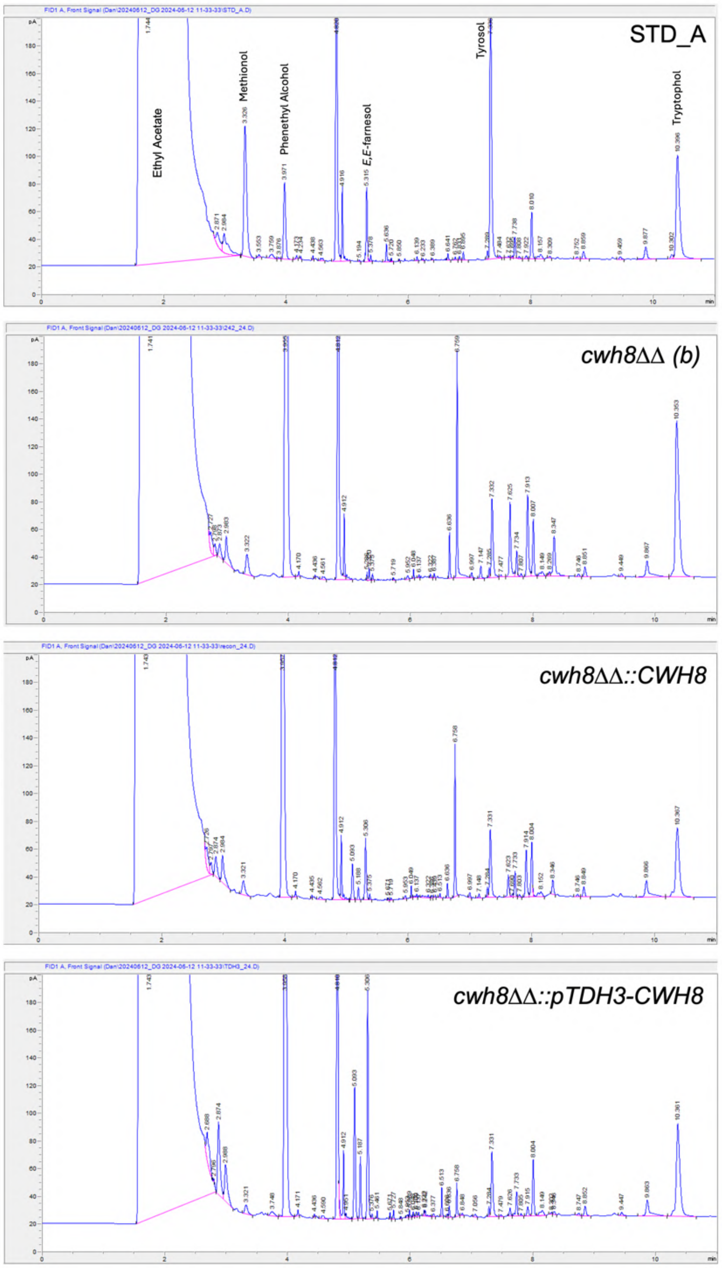

Figure S2. Representative gas chromatographs of ethyl acetate extracts a known standard (Standard A, Boone et al., 2022), *cwh8ΔΔ*., *cwh8ΔΔ*.::*CWH8* and *cwh8ΔΔ.*:*:pTDH3-CWH8* grown in YPD at 24 hours post-inoculation.

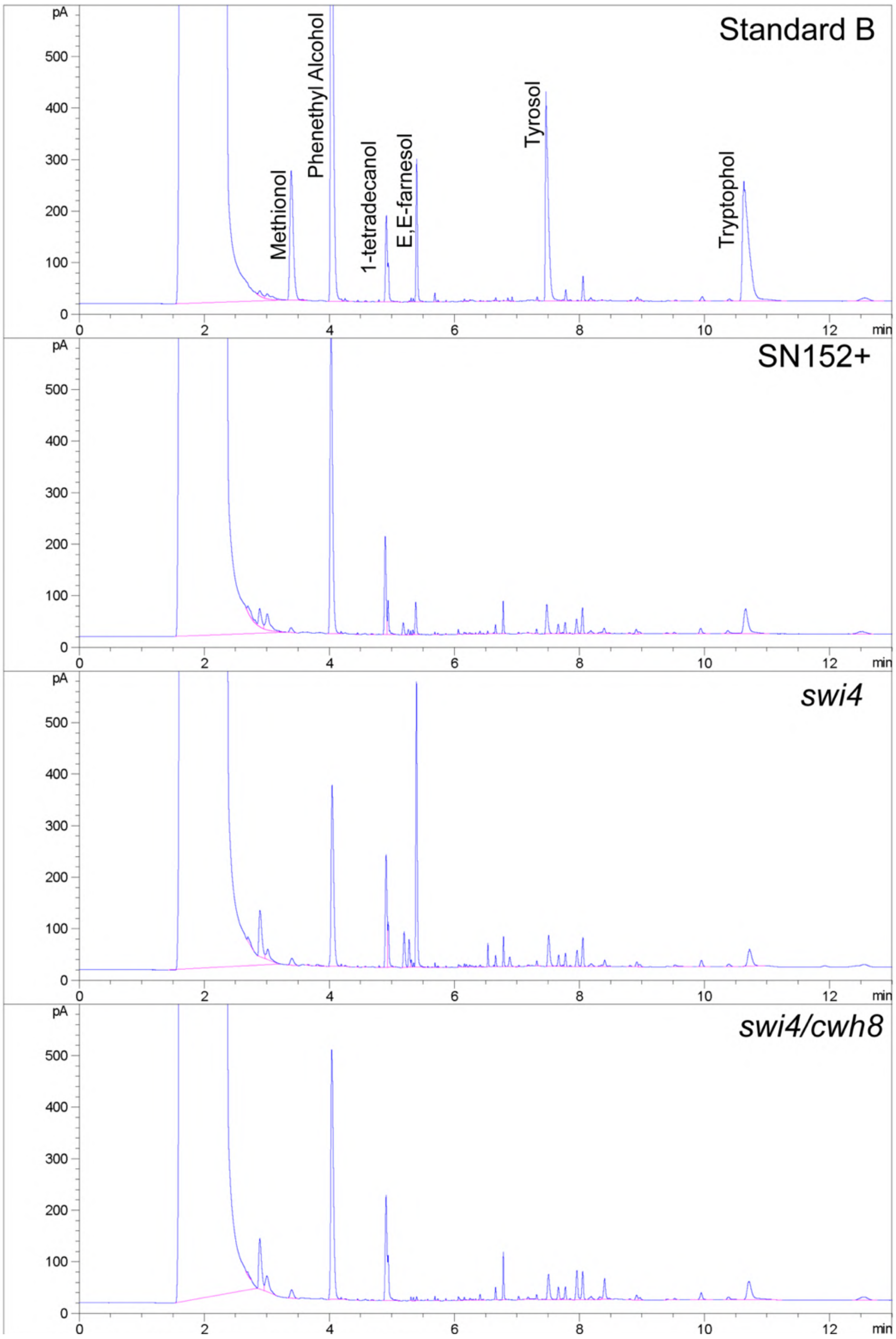

Figure S3. Representative gas chromatographs of ethyl acetate extracts a known standard (Standard B, Boone et al., 2022), SN152+, *swi4ΔΔ.*, and *swi4ΔΔ./cwh8ΔΔ.* grown in YPD at 24 hours post-inoculation.

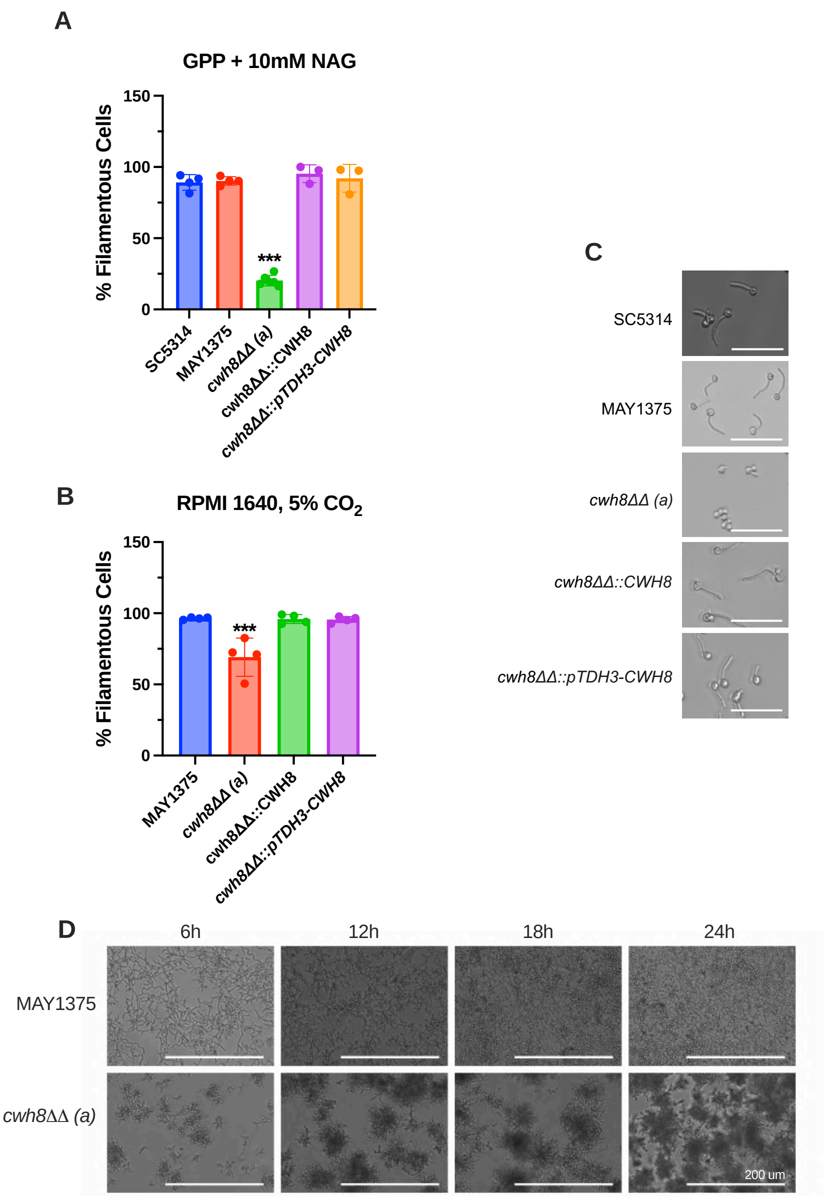

Figure S4. Filamentation and biofilm formation of the *cwh8ΔΔ* mutant is delayed. Resting cells were inoculated into either (A) GPP medium with 10 mM N-acetylglucosamine (NAG) at 37°C or (B) RPMI 1640 medium at 37°C, 5% CO_2 and incubated for 4 h. (C) Representative images of morphology at 4 h post inoculation in GPP + 10mM NAG medium. (D) Representative images of morphology at 6 , 12, 18, and 24 h post inoculation in GPP + 10mM NAG medium. Data are the mean percent of cells with germ tube ± SD of three independent experiments (n = 3).

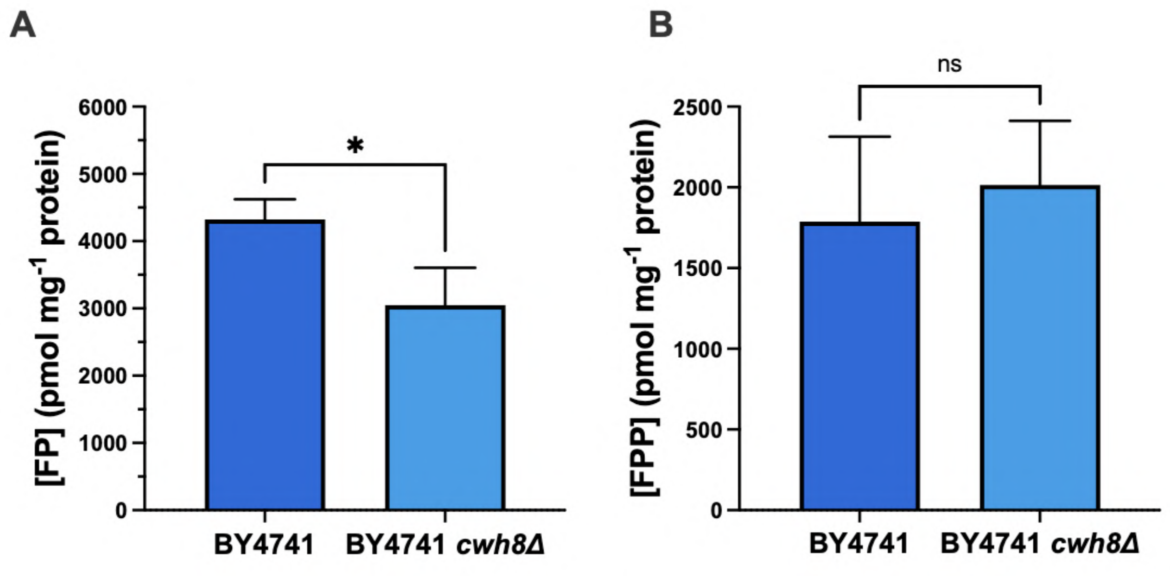

Figure S5. Quantitation of FP and FPP in the *S. cerevisiae cwh8*Δ (*cax4*Δ) mutant. (A) Intracellular farnesyl phosphate (FP) assessed at 24 hr post inoculation at 30°C in mRPMI-1640 by LC-MS/MS. (B) Intracellular farnesyl pyrophosphate (FPP) assessed at 24 hr post inoculation at 30°C in mRPMI-1640 by LC-MS/MS. Data are the mean concentration (pmol mg^{-1} protein ± SD of three independent experiments (n = 3).

A

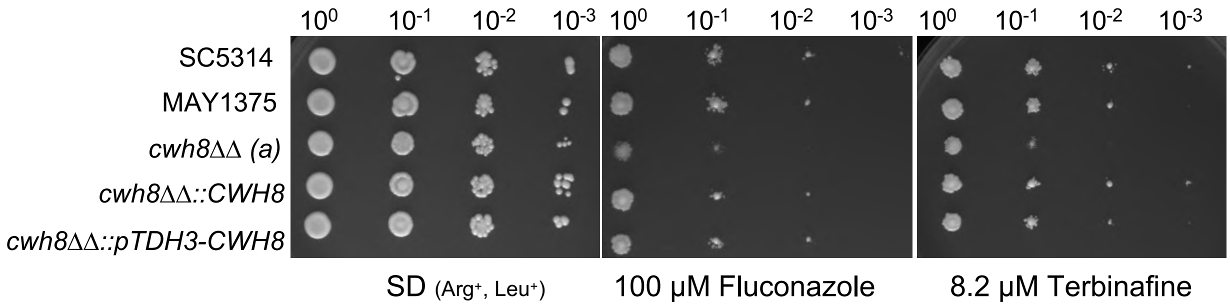

Figure S6. Susceptibility of MAY1375, *cwh8ΔΔ.*, *cwh8ΔΔ.*::*CWH8* and *cwh8ΔΔ.::pTDH3-CWH8* to fluconazole and terbinafine. (A) Serial dilution growth test on SD media (Arg^+, Leu^+) with the addition of either fluconazole (100 μM) or terbinafine (8.2 μM).
